# Magnesium and Diabetes: A Review of Recent Studies on the Efficacy of Supplementation with Mg in Humans Suffering from This Chronic Metabolic Disease

**DOI:** 10.3390/ijms27177620

**Published:** 2026-08-25

**Authors:** Agnieszka Ścibior, Manuel Aureliano, Zuzanna Romanowska, Lidia Radko, Tomasz Męcik-Kronenberg

**Affiliations:** 1 Laboratory of Oxidative Stress, Department of Biomedicine and Environmental Research, Institute of Biological Sciences, Faculty of Medicine, The John Paul II Catholic University of Lublin, Konstantynów St. 1J, 20-708 Lublin, Poland; 2 Faculdade de Ciências e Tecnologia (FCT), Campus de Gambelas, Universidade do Algarve, 8005-139 Faro, Portugal; 3 Centro de Ciências do Mar (CCMAR/CIMAR LA), Campus de Gambelas, 8005-139 Faro, Portugal; 4 Department of Paediatrics, Medical University of Warsaw, Żwirki i Wigury St. 63A, 02-091 Warsaw, Poland; zuzannamikus@gmail.com; 5 Department of Preclinical Sciences and Infectious Diseases, Faculty of Veterinary Medicine and Animal Sciences, Poznan University of Life Sciences, Wolynska St. 35, 60-637 Poznan, Poland; lidia.radko@up.poznan.pl; 6 Department of Pathomorphology, Faculty of Medical Sciences in Zabrze, Medical University of Silesia, 3 Maja St. 13, 41-800 Zabrze, Poland; patolog@interia.pl; 7 Collegium Medicum im. Dr. Władysław Biegański, Jan Długosz University, Washington St. 4/8, 42-200 Czestochowa, Poland

**Keywords:** diabetes, glycemic and insulinemic indices, humans, insulin resistance, magnesium

## Abstract

Diabetes mellitus (DM) is a chronic metabolic disease which, if left untreated or poorly controlled, can lead to serious complications. This global public health challenge is frequently associated with a deficit of Mg, which is one of the most important elements in the human body and is indispensable for many metabolic processes and the maintenance of human health. Due to its antioxidant and anti-inflammatory properties, its mitochondrial-supportive function, and its crucial role in the secretion and action of insulin as well as metal ion homeostasis, Mg may have great potential in the treatment of diabetes and diabetic complications. Therefore, we collected studies on a possible association between Mg and diabetes in humans. In other words, we intended to summarize data on the efficacy of oral Mg supplementation on glycemic and insulinemic parameters and Mg levels in biological specimens from humans with DM, gestational diabetes (GDM), insulin resistance (IR), and prediabetes (Pre-D). Our work indicates that, in some cases, supplementation with Mg may improve glycemic and/or insulinemic indices in hypo- or normomagnesemic individuals suffering from diabetes or IR. However, the results of certain studies did not show any beneficial effects of Mg intervention. Therefore, based on the findings presented in the current review, it can be concluded that further long-term studies on a larger population are required to demonstrate the efficacy of oral Mg supplementation in hypo- or normomagnesemic subjects with diabetes or at risk of developing the disease. It also seems justified to introduce routine determination of blood Mg levels (especially the ionized Mg form) in patients with diabetes and in those at risk of IR and glucose metabolism disorders. Moreover, it remains to be clearly determined which patients could benefit most from supplementation with this mineral, as its deficiency may impair insulin sensitivity and glucose uptake. To sum up, the results reviewed in the present report do not definitively indicate that supplementation with Mg may be regarded as a public health strategy for improving the outcomes of diabetes despite the fact that, in some cases, Mg has been reported to have a positive effect on the metabolic profile in subjects with diabetes and may be used as an adjuvant therapy of this modern-age disease.

## 1. Introduction

### 1.1. Diabetes: Selected Issues in a Nutshell

#### 1.1.1. Diabetes: Background

Diabetes mellitus (DM), a serious, chronic, and complex metabolic disorder with many long-term complications (including retinopathy, nephropathy, neuropathy, and increased risk of hypertension and coronary artery disease) [1,2], which is often accompanied by alterations in magnesium (Mg) status [3,4,5,6,7], is a global public health problem.

DM is one of the top 10 causes of disability in humans worldwide [8]. This condition is characterized by hyperglycemia caused by defects in insulin (INS) secretion, insulin action, or both [9]. Persistent hyperglycemia leads to progressive damage to various systems and tissues (especially cardiovascular, urinary, and nervous systems) and consequent short- and long-term complications (micro- and macrovascular complications included) [10]. There are various subtypes of diabetes. The most common are type 1 diabetes mellitus (T1DM) (representing 5–10% of diabetes cases), type 2 diabetes mellitus (T2DM) (the most common, with 90–95% of diabetes cases), and gestational diabetes mellitus (GDM) (occurring in 6–15% of pregnancies) [11].

#### 1.1.2. Diabetes Worldwide

Diabetes poses a serious threat to public health worldwide and has reached epidemic proportions. According to the International Diabetes Federation (IDF) Atlas, the top three countries with the highest estimated number of people living with T1DM in 2025 were the USA, India, and China, although India had the highest estimated number of people aged < 20 years living with T1DM [12]. In the same year, 9.5 million people were suffering from this disease globally, compared with 8.4 million in 2021 [13]. Regarding the epidemiology of specific subtypes, children are the population most affected by T1DM, as the incidence is the highest in this group. According to the IDF, in 2024, there were 219,000 children and adolescents (<20 years of age) diagnosed with this condition worldwide. Data provided by the same institution show that, in 2024, there were 589 million adults (20–79 years) suffering from diabetes globally, whilst more than 90% of these cases were T2DM [14]. According to Jessica L. Harding et al. [15], who conducted a systematic review of 32 countries and regions in 2022, the incidence of adult-onset T1DM demands further research, as existing data on the incidence of this condition are insufficient, especially from low- and middle-income countries.

In Poland, 3.1 million adults (20–79 years) struggled with diabetes in 2024. The most recent data indicate that about 80–90% of these cases were attributable to T2DM. Approximately 131,000 patients were suffering from T1DM [16]. According to a study from Wielkopolska, the incidence of childhood T1DM increased about 3.6-fold between 1998 and 2018, reaching 30.8 cases per 100,000 children annually, which is one of the most rapid increases in childhood T1DM incidence in Europe [17,18]. In Portugal, the *National Diabetes Observatory Annual Report—2024 Edition* estimated that the prevalence of diabetes among the Portuguese population aged from 20 to 79 years (8.1 million individuals) was 14.2% in 2024. This means that approximately 1.2 million Portuguese people in this age group have diabetes. The aging of the Portuguese population’s age structure (20–79 years) resulted in a 2.5 percentage point increase in the diabetes prevalence rate between 2009 and 2024, representing a growth of approximately 21.0% over this period. Regarding the composition of the diabetes prevalence rate, 56% of the individuals had already been diagnosed, while 44% remained undiagnosed [19].

#### 1.1.3. Type 1 and Type 2 Diabetes Mellitus

T1DM is an autoimmune disorder resulting in the gradual and complete destruction of insulin-producing β-cells in the pancreas by autoreactive T lymphocytes. So-called islet antibodies may be present and precede the symptoms of the disease by many years. As they target various β-cell antigens, they lead to hyperglycemia and severe insulin deficiency. At the time of diagnosis, usually around 60–90% of β-cells are destroyed and from this point on patients require lifelong insulin therapy [20]. Genetic susceptibility to the disease is associated with the presence of human leukocyte antigens (HLAs) of which HLA-DR and HLA-DQ are the most highlighted for being responsible for 50% of the inherited risk of developing T1DM [21,22]. This type of diabetes has an early onset, usually affecting children and young adolescents, although it may occur at any age [23]. Quick diagnosis and proper treatment help prevent short- and long-term complications. Management is mainly based on insulin therapy and glycemic control, as mentioned above; however, proper dietary treatment and patient education are also pivotal. Altogether, these measures prevent acute complications, i.e., hypoglycemia and diabetic ketoacidosis [24].

T2DM is based on two mechanisms: IR in peripheral tissues and impaired insulin secretion by pancreatic β-cells [25]. IR manifests as excessive glucose production in the liver and impaired glucose uptake in muscle, hepatic, and adipose tissue. These abnormalities lead to hyperinsulinemia, a condition in which β-cells become increasingly inefficient in producing sufficient amounts of insulin to overcome the condition and fail to maintain adequate blood glucose levels. Altogether, these processes contribute to hyperglycemia and T2DM development [26]. T2DM is strongly linked to modifiable risk factors such as obesity, sedentary lifestyle, and poor dietary habits, all of which promote the development of IR [27]. Nevertheless, some patients may have a genetic predisposition [28]. It is also more prevalent in some populations, with Japanese, Hispanic, and Native American individuals being at the greatest risk of developing the disease [29,30,31]. Treatment for this type of diabetes consists of lifestyle modification, pharmacological therapy (metformin, glucagon-like peptide-1 (GLP-1) receptor agonists, sodium-glucose co-transporter 2 (SGLT-2) inhibitors, statins, anti-inflammatory drugs, and insulin therapy) [32], and management of comorbidities. The aforementioned measures help to achieve glycemic control, prevent complications, and reduce cardiovascular and renal risk [33,34,35].

#### 1.1.4. Gestational Diabetes Mellitus

GDM refers to any extent of glucose intolerance detected during pregnancy [36]. Its usual onset occurs in the second or the third trimester and is driven by relative insulin deficiency due to pregnancy-induced IR and placental hormones following impaired response to blood glucose levels [37,38]. Women in advanced maternal age (i.e., 35 years old and older) and those with polycystic ovary syndrome (PCOS), obesity, or a family history of diabetes are the most commonly affected patients [39,40]. This condition threatens both the mother and the developing fetus. Preeclampsia, polyhydramnios, or delivery-related complications such as an increased risk of cesarean section, instrument-assisted vaginal birth, and perineal trauma resulting from fetal macrosomia [41] are among some common maternal complications. Postpartum, women with GDM may also be affected by obesity [42], development of cardiovascular disease, T1DM, T2DM [43,44], hypertension, dyslipidemia [45] or even a higher risk of pancreatic cancer [46]. Fetal complications include a high risk of macrosomia, impaired eye and brain development [41], birth injury, shoulder dystocia, fetal death, perinatal asphyxia [47], respiratory distress syndrome [48], hypoglycemia [49], hyperinsulinemia, and obesity later in life [36].

#### 1.1.5. Insulin Resistance

IR is a metabolic disorder characterized by a diminished sensitivity of peripheral tissues—particularly skeletal muscle, the liver, and adipose tissue—to the actions of insulin. As a result, higher circulating insulin levels are required to maintain normal glucose regulation. Initially, pancreatic β-cells compensate through increased insulin secretion; however, prolonged compensation may ultimately result in β-cell failure and the development of T2DM [50]. Beyond its well-established role in the development of diabetes, IR also contributes to the pathogenesis of several disorders, including cardiovascular disease, non-alcoholic fatty liver disease (NAFLD), hypertension, dyslipidemia, PCOS, metabolic syndrome, and tumors. The key mechanisms of the phenomenon include low-grade inflammation, oxidative stress, mitochondrial dysfunction, ectopic lipid accumulation, visceral adiposity, and defects in insulin signaling and glucose transporter type 4 (GLUT4) translocation [51].

#### 1.1.6. Risk Factors for Diabetes

Diabetes is a disease with a multifactorial etiology. While T1DM and T2DM share a number of common risk factors, some are more characteristic of one disease type than the other, reflecting differences in their underlying pathophysiology. One of the main risk factors for T1DM is genetic predisposition, with a crucial role of the HLA-DR3/DR4 genotype and a family history (first-degree relatives, twins). Environmental triggers such as viral infections, particularly those caused by enteroviruses (especially Coxsackie B virus) [52], or exposure to toxins and/or chemical compounds also contribute to the development of the disease. The presence of islet autoantibodies is a significant predictor of progression to T1DM. There also exists a relationship between micronutrient deficiencies and disease onset, especially a lack of vitamin D, vitamin E, or zinc either during pregnancy or later in childhood. Other frequently listed risk factors include gut microbiome alterations, excessive consumption of a “Western” diet, obesity, higher birth weight, increased height growth, perinatal factors, and seasonality. Some data suggest a link between T1DM and early-childhood exposure to gluten or cow’s milk; however, this topic requires further research [53]. Risk factors for T2DM include genetics, sex (men are more predisposed than women), age (T2DM incidence increases with age), and ethnicity. Consequences of unhealthy habits such as excessive body weight, visceral adiposity, low physical activity, and a poor diet are other significant determinants of the disease. T2DM is also associated with many comorbidities, including hypertension, dyslipidemia, non-alcoholic fatty liver disease (NAFLD), IR, prediabetes, and metabolic syndrome. Other important contributors are GDM, depression, a history of drug treatment, smoking, alcohol consumption, and vitamin D and magnesium deficiencies [54,55].

#### 1.1.7. Complications of Diabetes

The crosstalk between genetic and environmental factors, as well as among organs (the kidney, heart, brain, adipose tissue, liver, skeletal muscle, pancreas, and intestine), plays a role in the pathogenesis of diabetes and its adverse outcomes [56]. Hyperglycemia gives rise to endothelial dysfunction, atherosclerosis [57], oxidative stress, inflammation [58,59], metabolic disturbances, and mitochondrial dysfunction [60]. The complications are often divided into microvascular and macrovascular [61]; however, this classification represents a major simplification, as these pathologies share underlying mechanisms, signaling pathways, and interactions [62,63,64,65,66]. Microangiopathy is an underlying mechanism of the development of diabetic retinopathy [67], diabetic kidney disease, diabetic neuropathy (peripheral, sensory, autonomic, and motor), diabetic cardiomyopathy, and diabetic foot disease [68]. Macroangiopathies include peripheral arterial disease, diabetic encephalopathy (e.g., ischemic stroke, transient ischemic attacks, vascular dementia, and neurodegenerative changes), and diabetes-related heart disease [69]. Metabolic dysfunction-associated steatotic liver disease (MASLD) and metabolic dysfunction-associated steatohepatitis (MASH), in general labeled as diabetic hepatopathy, are also metabolic complications of the disease [70]. Other complications include restrictive lung disease [71], susceptibility to infection, or impairment of cytokine production [72]. Notably, there are disorders coexisting with T1DM and T2DM or sharing underlying mechanisms. T1DM is often associated with such autoimmune diseases as celiac disease, rheumatoid arthritis, Addison’s disease, and autoimmune thyroid disease [73,74,75], whereas Alzheimer’s disease [76], PCOS [77], Cushing’s syndrome [78], and pancreatic cancer [79] are closely linked to T2DM.

### 1.2. Magnesium: Selected Issues in a Nutshell

#### 1.2.1. Role of Mg in Biological Systems

**Figure 1:**
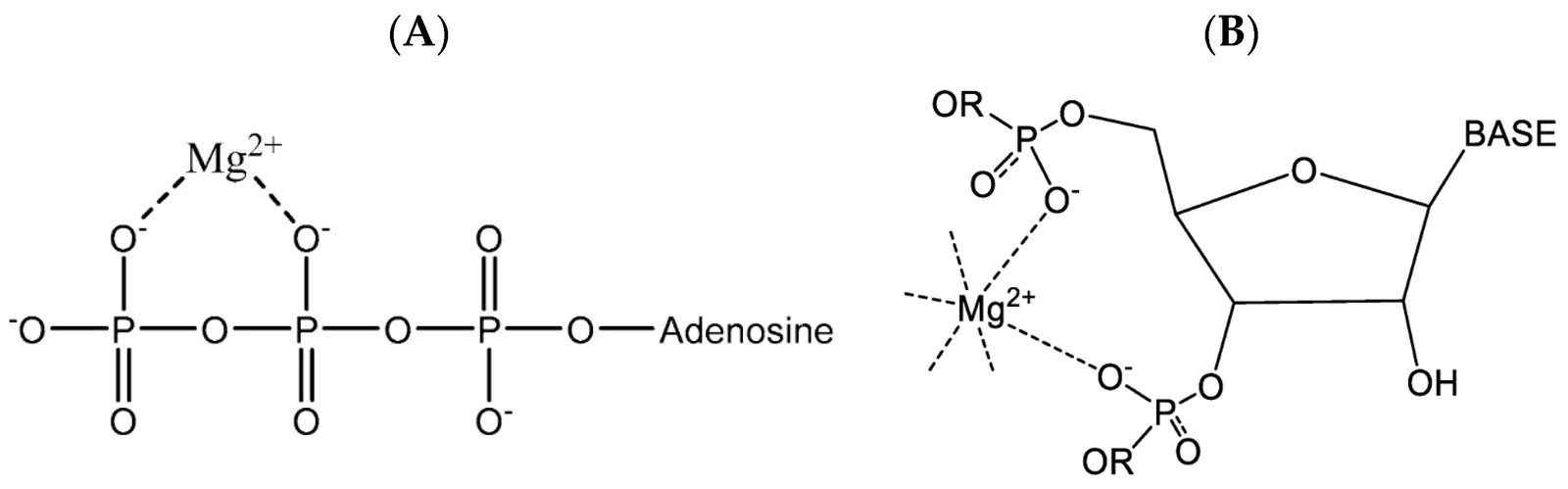
The primary function of Mg^2+^ in biological systems includes electrostatic activation of substrates and stabilization of nucleic acids, such as (**A**) ATP and (**B**) DNA. Reproduced from Refs. [83,84] with permission from MDPI.

Mg, the most abundant intracellular divalent cation present in all living cells, is an essential element for human health. It plays relevant biological roles in biological systems, as it may form complexes with ATP, namely MgATP (Figure 1A), which is the principal energetic substrate needed for many essential processes in organisms and cells, such as muscle contraction, active transport, and intracellular signaling [80]. The geometry of Mg in the Mg-ATP -complex is octahedral [81]. In fact, the Mg-ATP -complex is abundant in all forms of life, with 90% of intracellular ATP existing in this form [82]. This bioelement induces a conformational rearrangement in ATP and ADP to enable catalysis [82]. Moreover, Mg^2+^ is responsible for the stabilization of nucleic acid structures through electrostatic interactions with O^−^ from phosphate (Figure 1B).

**Figure 2:**
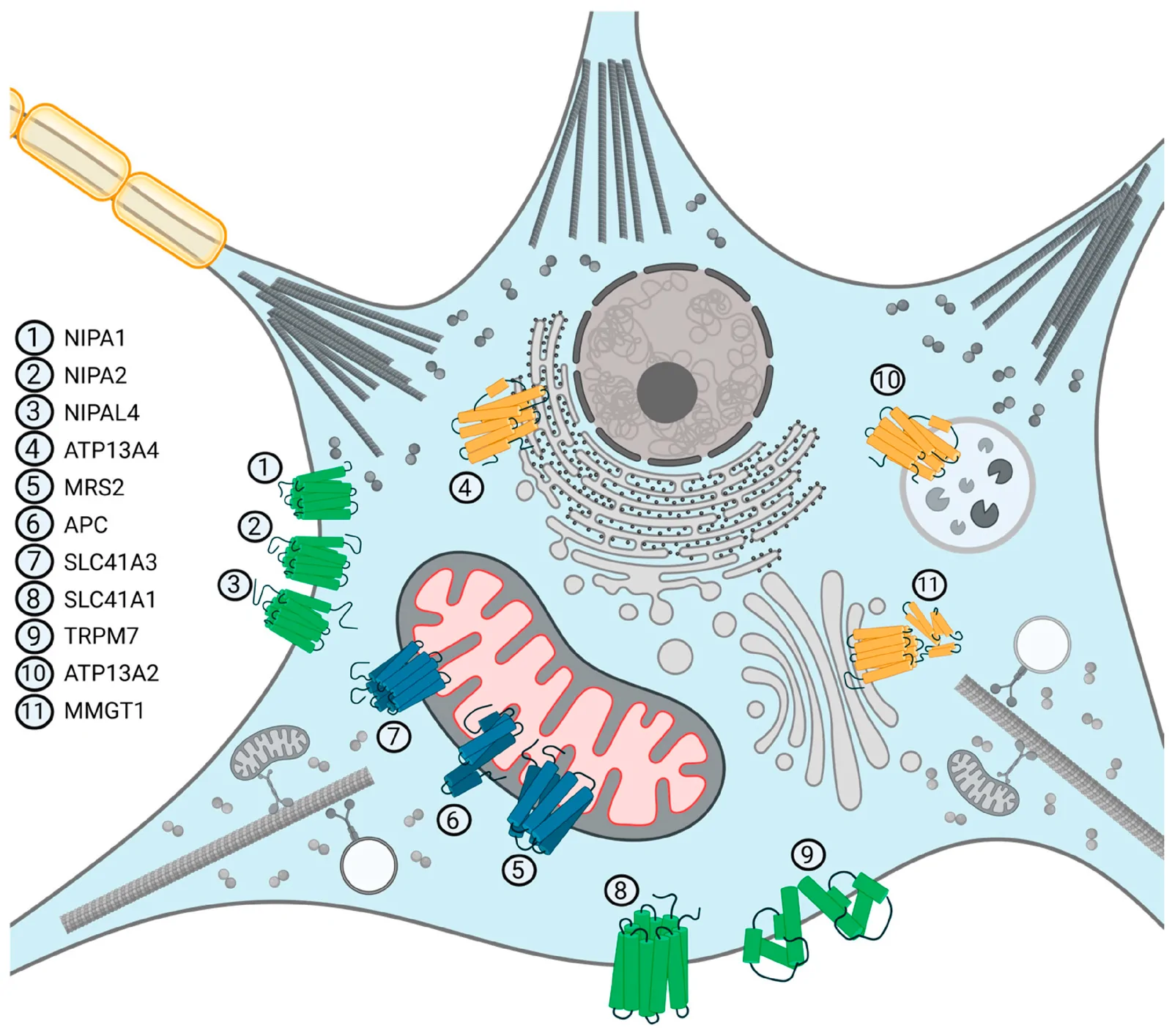
Magnesium transporters localized in the subcellular structures of neurons. Reproduced from Ref. [90] with permission from MDPI. APC: adenomatous polyposis coli protein; ATP13A2: cation-transporting ATPase 13A2; ATP13A4: cation-transporting ATPase 13A4; MMGT1: membrane magnesium transporter 1; MRS2: mitochondrial RNA splicing 2 protein; NIPA1: NIPA magnesium transporter 1; NIPA2: NIPA magnesium transporter 2; NIPAL4: NIPA-like domain-containing 4; SLC41A1: solute carrier family 41 member 1; SLC41A3: solute carrier family 41 member 3; TRPM7: transient receptor potential melastatin type.

Several metals can replace and/or substitute Mg in nature. For instance, Cu and Zn are able to replace Mg in the center of the porphyrin ring and inactivate chlorophyll [85]. In contrast to Ca^2+^, relatively few metalloproteins contain Mg^2+^, for example ribonucleases, enolases, and pyruvate carboxylases, although around 70% of the body’s enzymes require Mg to function properly [86]. Intracellular Mg^2+^ concentrations differ considerably from those of Ca^2+^: the pCa value is 9, whereas the pMg value is 3 [87]. Although first described for Ca^2+^, Mg^2+^ homeostasis is strictly controlled by Mg^2+^ channels and transporters [88]. Cellular Mg^2+^ transport occurs mainly through active transport [89]. There are three major players involved in Mg^2+^ influx: the transient receptor potential cation channel, subfamily M, member 6 (TRPM6); Mg transporter 1 (MagT1); and the cyclin M (CNNM) transporter family. The most important transporter responsible for transport out of the cell is solute carrier family 41 member 1 (SLC41A1) (Figure 2). As illustrated below, the chanzyme TRPM7 and transporters NIPA1, NIPA2, NIPAL4, and SLC41A1 belong among cell membrane proteins. ATP13A4 is localized in the endoplasmic reticulum membrane, ATP13A2 in the lysosomal membrane, and MMGT1 in the Golgi apparatus membrane. Transporters MRS2, APC, and SLC41A3 are mitochondrial membrane proteins.

**Figure 3:**
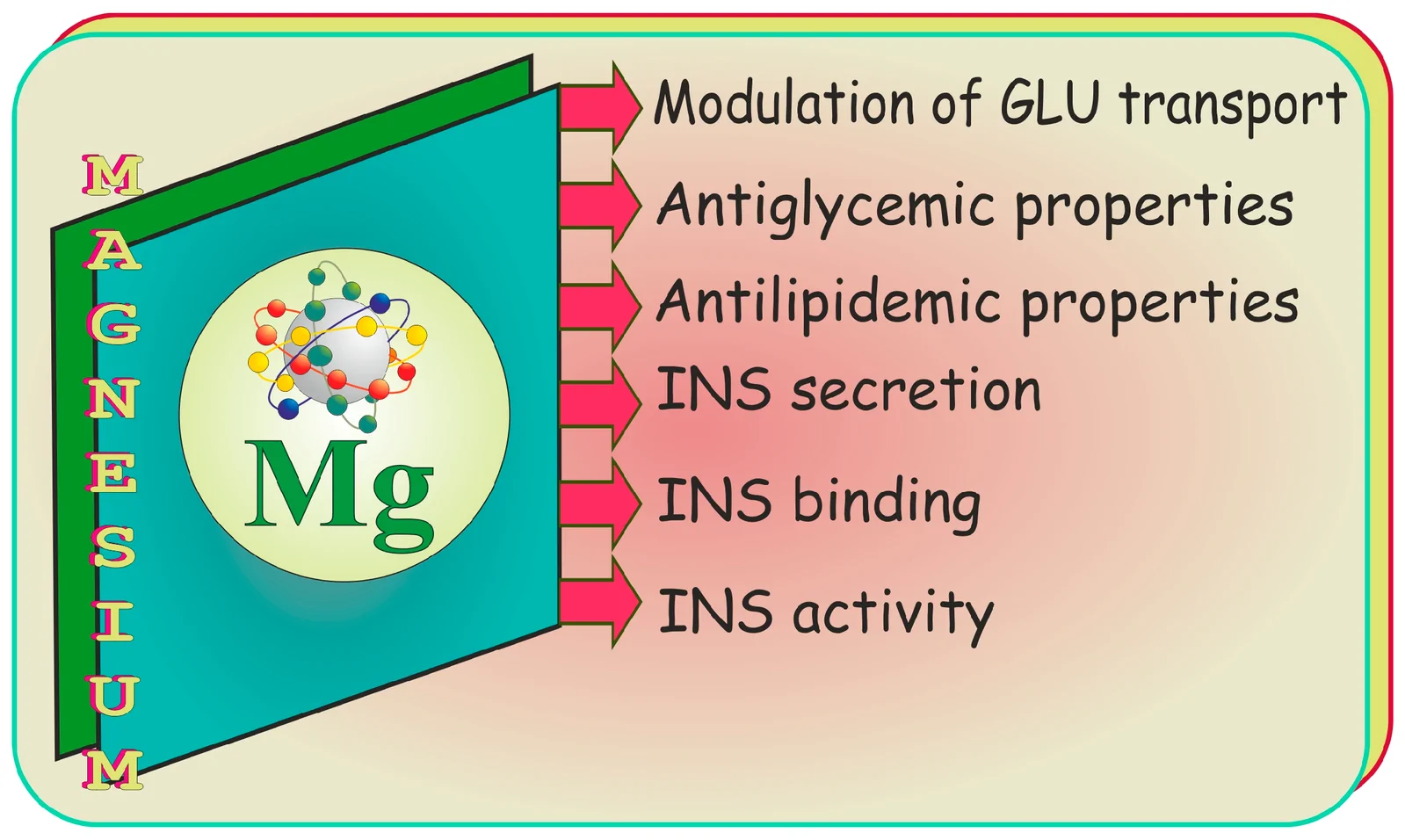
Summary of the role of Mg with respect to carbohydrate and lipid metabolism—based on the available literature cited in Section 1.2.1. GLU: glucose; INS: insulin.

Mg plays a fundamental role in various metabolic pathways involving carbohydrates and lipids [91]. This macronutrient exerts several beneficial effects, including antiglycemic and antilipidemic properties [92]. It also affects the release, binding, and activity of insulin (INS) [91]. Additionally, it plays a key role in regulating glucose (GLU) transport [93]. A brief summary of the functions of Mg with respect to carbohydrate and lipid metabolism is presented in Figure 3.

#### 1.2.2. A Brief Historical Framework Related to Diabetes and Mg on the Timeline

**Figure 4:**
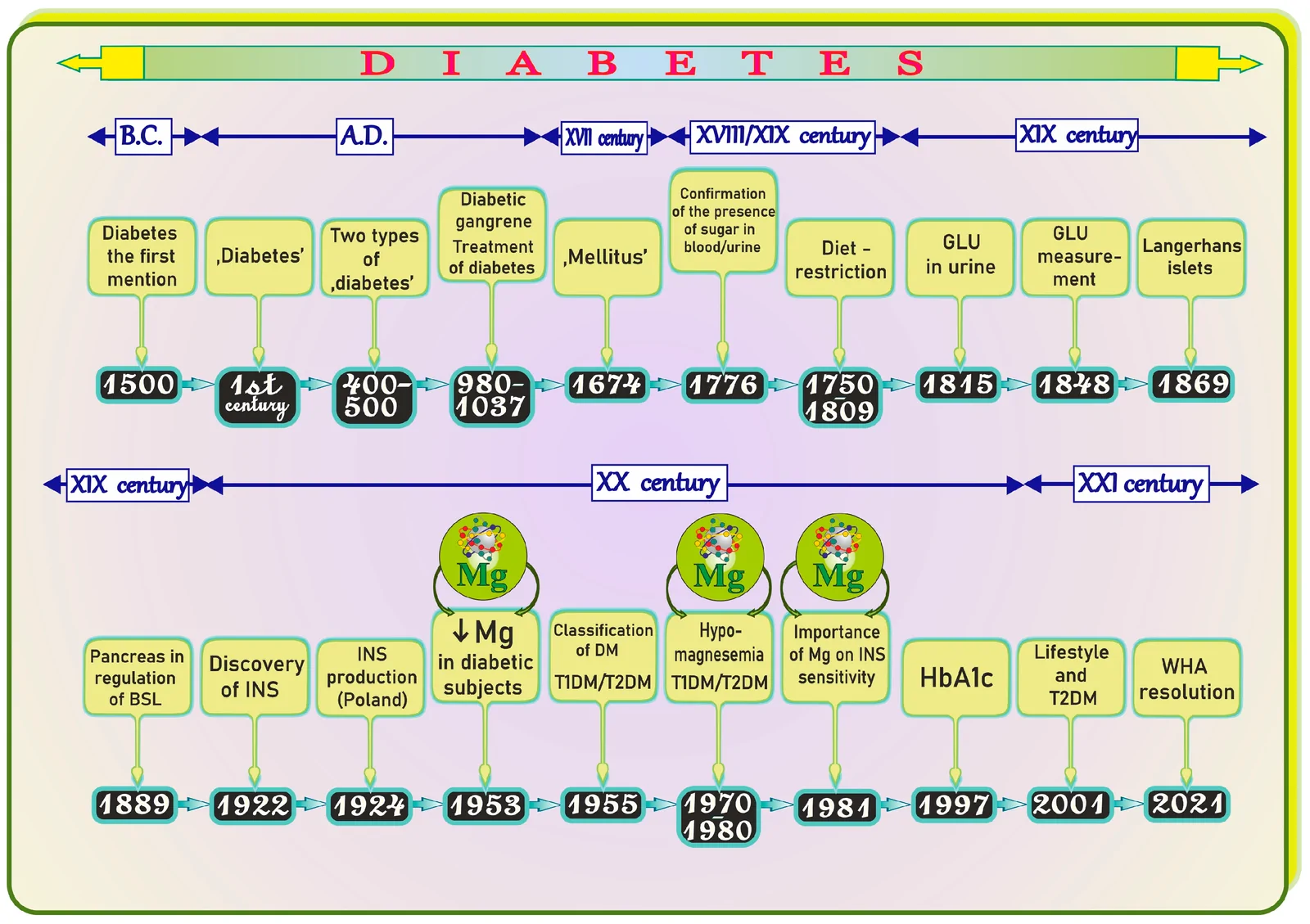
Historical view of diabetes—based on the available literature cited in Section 1.2.2. BSL: blood sugar level, HbA1c: glycosylated hemoglobin, GLU: glucose, INS: insulin, Mg: magnesium, WHA: World Health Assembly. ↓: reduction.

The aim of this section is to present the historical background of studies on diabetes and Mg in a nutshell to draw the reader’s attention to important events associated with this essential bioelement and one of the most common chronic life style-related diseases in terms of time. The description provided in this section is accompanied by Figure 4, in which selected points are presented in chronological order for the reader’s convenience.

Diabetes was first mentioned in ancient Egyptian manuscripts in 1500 B.C. [94]. At that time, doctors observed excessive urine excretion resulting in general wasting of the body, and then death. In the first century A.D., the Greek physician Aretaeus of Cappadocia first used the term ‘diabetes’ to describe a disease associated with excessive fluid intake and increased urination [95]. In 400–500 A.D., Indian doctors Sushruta and Charaka (who lived around 400–500 A.D.) identified two types of diabetes, which today are referred to as T1DM and T2DM. In 980–1037 A.D., the Persian physician and philosopher Avicenna, called the father of modern medicine, described diabetic gangrene and tried to treat diabetes with a mixture of lupine grains, fenugreek, and turmeric [94].

In 1674, the British physician Thomas Willis was probably the first to use the term ‘mellitus’ (Latin: ‘sweet like honey’) [96], which was added to the name ‘diabetes’. He diagnosed diabetes by examining patients’ urine, which he described as ‘honey-like’. In 1776, Matthew Dobson (an English physician) was the first to confirm the presence of excess sugar in urine and blood as a cause of their sweetness [96]. In 1750–1809, a Scottish military surgeon, John Rollo, suggested the use of the first diet based on restriction of carbohydrates in diabetic patients [96]. In 1815, the French chemist Michel-Eugène Chevreul identified the sugar present in patients’ urine as glucose (GLU) [96]. In 1848, the German pharmacist and chemist Herman Christian von Fehling developed a method for measuring glucose levels [96]. Further, in 1869, Paul Langerhans, who was working on his doctoral dissertation, identified cells that are known as the islets of Langerhans [94]. In 1889, two German medical researchers Joseph von Mering and Oskar Minkowski, who conducted a series of experiments on dogs, demonstrated that pancreatectomized animals developed diabetes [94,96]. They were the first to describe the role of the pancreas in regulating blood sugar levels, but they were unable to isolate the substance responsible for this activity. In 1922, Frederick Banting and Charles Best discovered insulin (INS) [95]. In 1924, Kazimierz Funk, a Polish researcher and creator of the term ‘vitamin’, organized the production of INS from beef pancreas in Poland [97]. In 1953, reduced blood Mg levels in diabetic individuals were documented and a possible relationship between DM and hypomagnesemia was first suggested [98]. In 1955, a method of testing INS levels in the body was developed, and classification into Type 1 (INS-dependent) and Type 2 (INS-independent) diabetes was officially introduced into science [97]. In the early 1980s, i.e., in 1981, the importance of Mg on INS sensitivity was suggested [99]. In the 1970s–1980s, hypomagnesemia was frequently noted in both T1DM and T2DM [3,100,101,102,103,104]. In 1997, glycated hemoglobin (HbA1c) was discovered and introduced as a standard method of measuring diabetes control [105]. In 2001, Tuomilehto published the results of studies clearly indicating that changing the lifestyle and diet, as well as increasing physical activity may successfully reduce the incidence of T2DM [97]. Finally, in 2021, the World Health Assembly (WHA), i.e., the highest decision-making body of the World Health Organization (WHO), agreed on a resolution to strengthen the prevention and control of diabetes [97].

## 2. Absorption, Distribution, and Renal Handling of Mg

**Figure 5:**
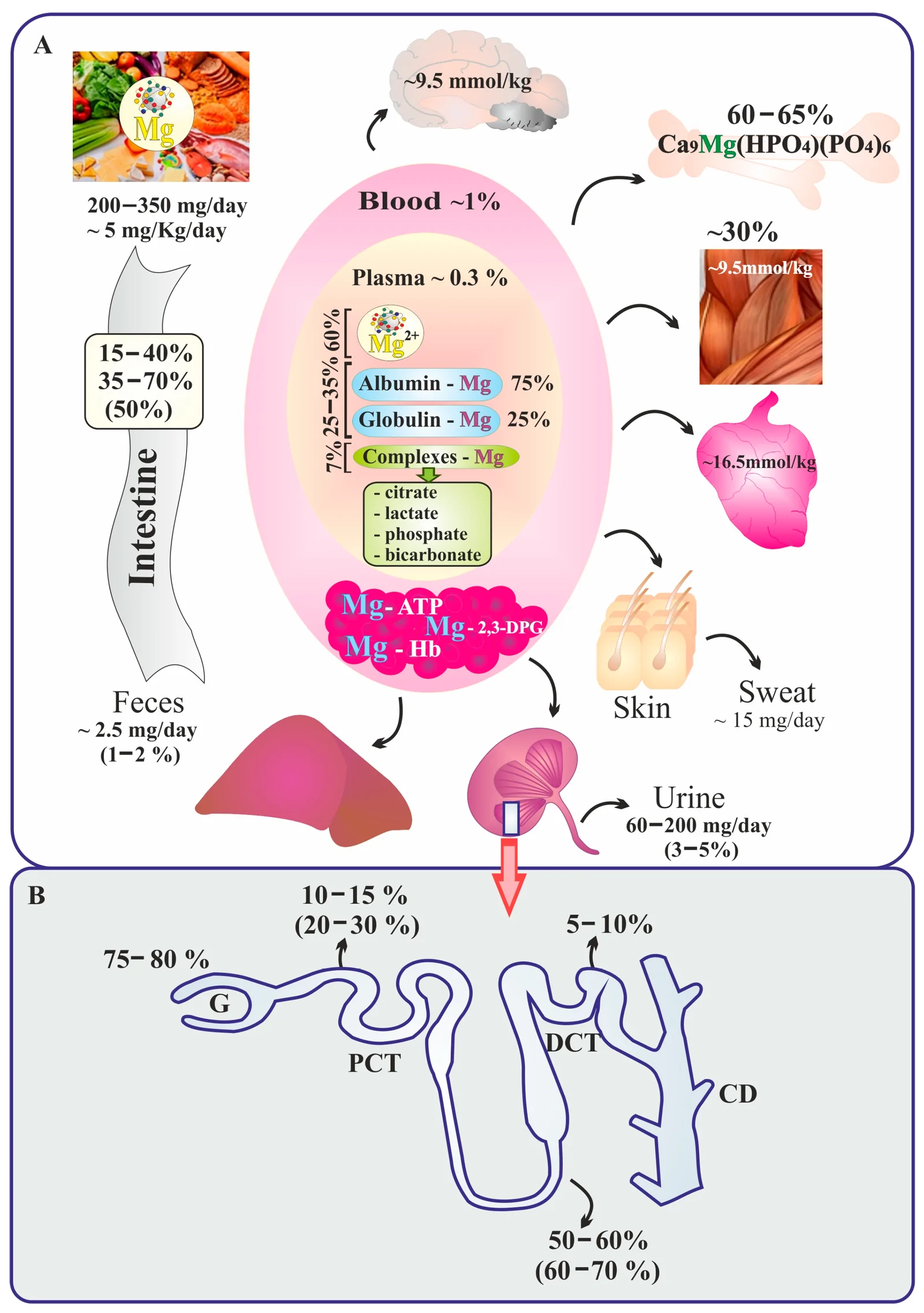
Absorption and distribution of Mg (**A**) and reabsorption of Mg along the nephron (**B**)—based on the available literature cited in Section 2. CD: collecting duct; DCT: distal convoluted tubule; G: glomerulus; Hb: hemoglobin; PCT: proximal convoluted tubule; 2,3-DPG: 2,3-bisphosphogluconate.

The absorption, distribution, and reabsorption of Mg along the nephron are summarized in Figure 5. The homeostasis of this macronutrient (g/Kg) is tightly regulated and depends on the balance between intestinal absorption and renal reabsorption and excretion [106]. Less than 1% of total body Mg is present in the blood, with a larger portion present in erythrocytes (RBC), where it is bound to ATP, hemoglobin (Hb), and 2,3-bisphosphogluconate (2,3-DPG) [107,108]. Most of the body’s Mg, i.e., about 60–65%, resides in bones as hydroxyapatite Ca_9_Mg(HPO_4_)(PO_4_)_6_, 30% in muscles, 19.3% in soft tissues, 0.5% in RBCs, and 0.3% in extracellular fluid [107,109,110]. In plasma, Mg is present in three forms: about 60% of Mg is available as ionized Mg^2+^, or is free, while the rest is complexed to anions such as citrate, lactate, phosphate, and bicarbonate (around 15%) and to proteins such as albumin and globulin (around 25–35%). Of the protein fraction, 25% is bound to albumin and 8% is bound to globulin [106,107,108]. Ionized Mg (Mg^2+^) is considered the biologically active form taking part in enzymatic reactions and physiological processes [106]. Bone and liver tissues act as major Mg reservoirs in the body [106]. In the kidney, which plays a major role in Mg homeostasis and the maintenance of plasma Mg concentration [111], approximately 75–80% of total plasma Mg is filtered at the glomeruli, of which around 95% is reabsorbed along the nephron. Around 10–15% (20–30%) is reabsorbed in the proximal convoluted tubule (PCT), 50–60% (60–70%) in the cortical thick ascending limb of the loop of Henle (TAL), and 5–10% in the distal convoluted tubule (DCT) [106,107,109,112,113,114]. A normal dietary Mg intake is approximately 200–350 mg/day and depends on the Mg concentration in drinking water and the food composition [111]. In healthy individuals, about 30–50% of ingested Mg is absorbed in the intestine, which can increase up to 80% during deficiency of this bioelement [106]. Most of the absorption process occurs in the ileum and colon. The level of Mg absorption varies depending on endogenous Mg status [111]. It is estimated that, under normal conditions, the daily excretion of Mg via feces and urine is about 1–2% and 3–5%, respectively. When Mg stores are normal, excretion usually equates with absorption [112]. As mentioned in the Introduction, 90% of Mg^2+^ exists in the form of Mg-ATP. However, millimolar intracellular Mg^2+^ concentrations can be found in cells, with pMg of pMg(serum) = −log (Mg) = 3.0706 [115]. The Mg requirement for healthy adults is estimated at 300–400 mg/day [116].

**Figure 6:**
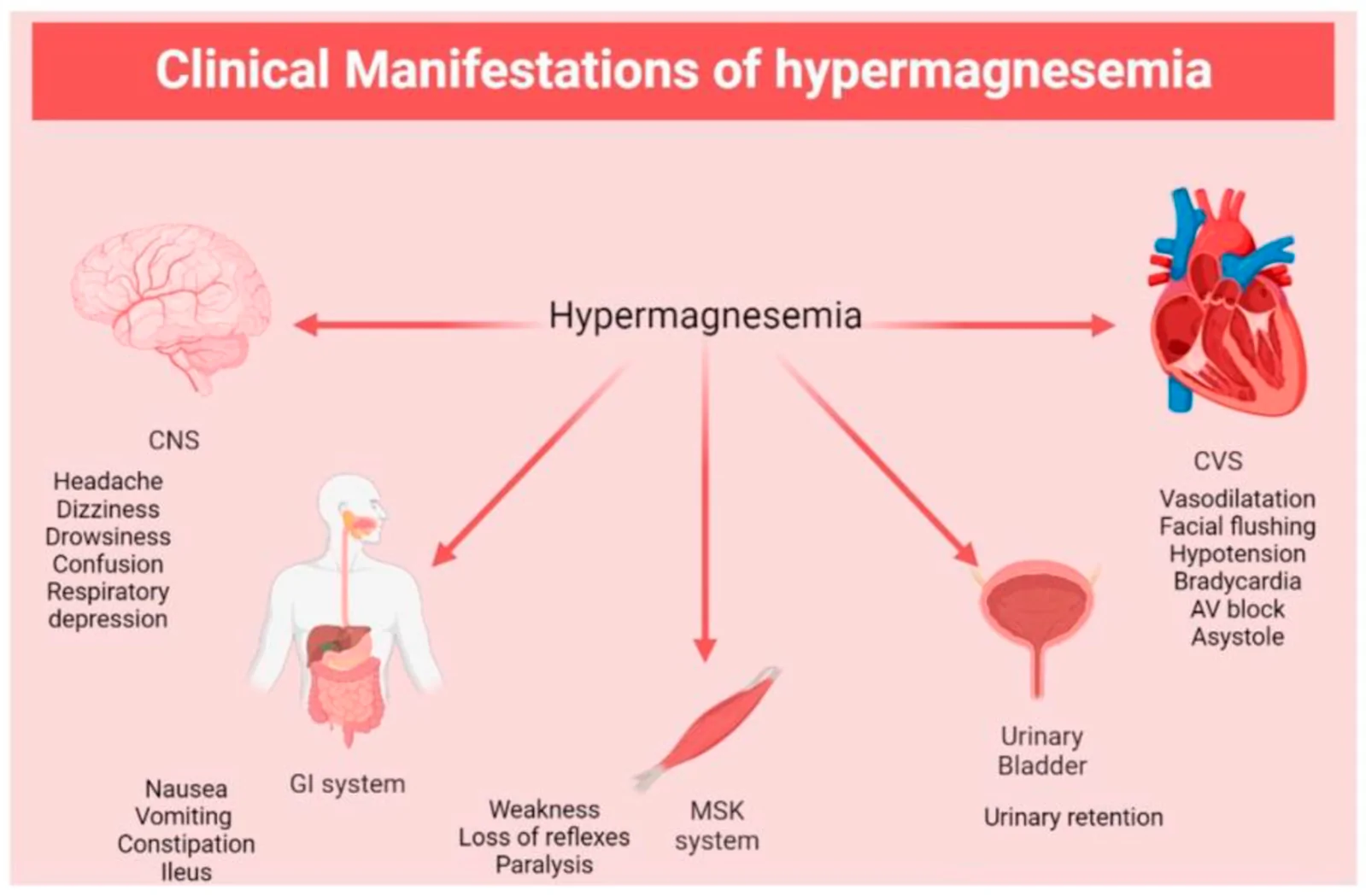
Overview of the effects of hypermagnesemia. Reproduced from Ref. [83] with permission from MDPI.

Several pathologies are associated with deficiency and/or excess of Mg, which has attracted considerable interest due to its potential role in the pathogenesis of certain illnesses [116,117,118]. Importantly, dysregulation of the homeostasis of this macroelement is involved in various cellular malfunctions and diseases, including cardiovascular disease, hypertension, obesity, Parkinson’s disease, cancer, and T2DM [119,120,121]. In fact, hypomagnesemia has been observed in the majority of patients with heart failure and T2DM, and Mg supplementation has been reported to improve cardiac function and IR [122]. More information about Mg^2+^ deficiency, which may have multiple causes [123], can be found in the next chapter of this review. In contrast, hypermagnesemia, i.e., an Mg^2+^ excess, is a rather rare condition owing to effective renal excretion [124]. When it appears, it is usually observed in patients with kidney dysfunction. Its symptoms vary from nausea and headaches to severe and dangerous complications affecting the central nervous system (CNS) and cardiovascular system (CVS) (Figure 6).

## 3. Hypomagnesemia and Lifestyle-Related Diseases Including Diabetes and Obesity—A Brief Outline

**Figure 7:**
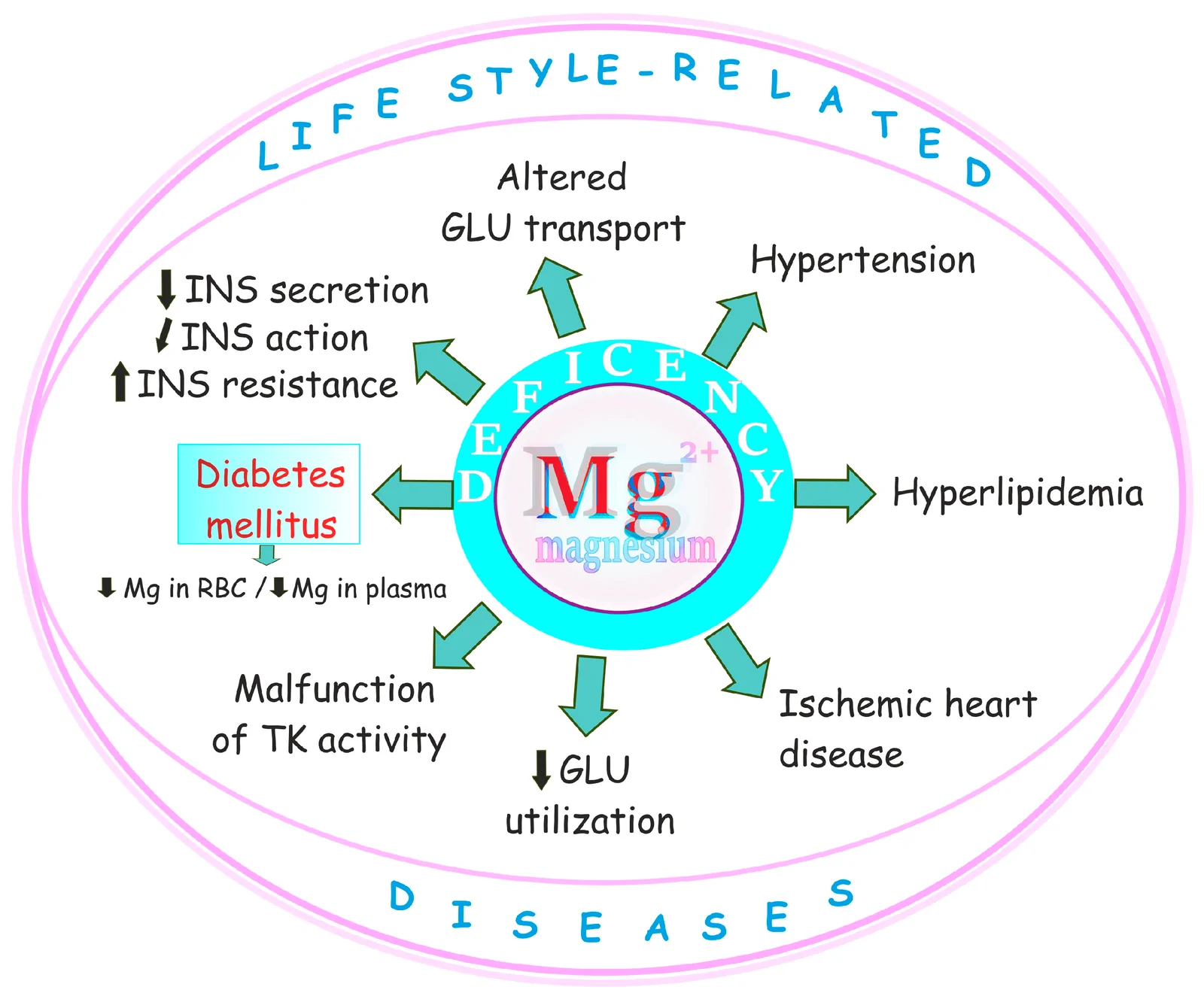
Selected manifestations of Mg deficiency—based on the available literature cited in Section 3. GLU: glucose; INS: insulin; RBC: erythrocytes; TK: tyrosine kinase. ↓: decrease; ↑: increase; ↙: impairment.

The key role of Mg in energy metabolism and signal transduction as well as modulation of the transport function and activities of enzymes (especially those that use nucleotides as cofactors or substrates) makes Mg deficiency a potential health hazard. It has been reported that the deficit of this bioelement is linked to lifestyle-related diseases, such as DM [125,126], hyperlipidemia [92], hypertension [126], and cardiovascular abnormalities [126]. Mg deficiency has also been hypothesized to be involved in an increase in IR [127,128,129] and in a decrease in INS secretion [130]. In addition, decreased Mg levels have been associated with malfunction of tyrosine-kinase (TK) activity, impairment of the action of INS, which has been proposed as a regulatory hormone of Mg balance [131], alteration of GLU transport, and reduction in GLU utilization [130]. Furthermore, it has been reported that the accumulation of Mg in erythrocytes (RBC), i.e., the primary GLU-consuming cells [132], in patients with T1DM and T2DM was impaired, in comparison with that in control subjects [4,133]. Moreover, in diabetic individuals with T1DM or T2DM, plasma/serum Mg levels, which are commonly measured and reflect only a small part of the total body Mg content, have been found to be reduced as well [3,5,6,7,103,134,135,136]. It has also been observed that the majority of T2DM patients have hypomagnesemia, and Mg supplementation has improved IR [122]. Additionally, patients with hypomagnesemia exhibit a more rapid disease progression and have an increased risk for diabetes complications [137]. Thus, Mg deficiency has been recognized as a common problem in both types of diabetes. In addition, the presence of this bioelement has been noted to be inversely correlated with the level of glycemic control and the development of complications [103,104,138]. Details on the levels of Mg in the blood of subjects with hypo- or normomagnesemia and diabetes are presented in a later part of this review. Selected clinical manifestations of hypomagnesemia are briefly summarized in Figure 7.

**Figure 8:**
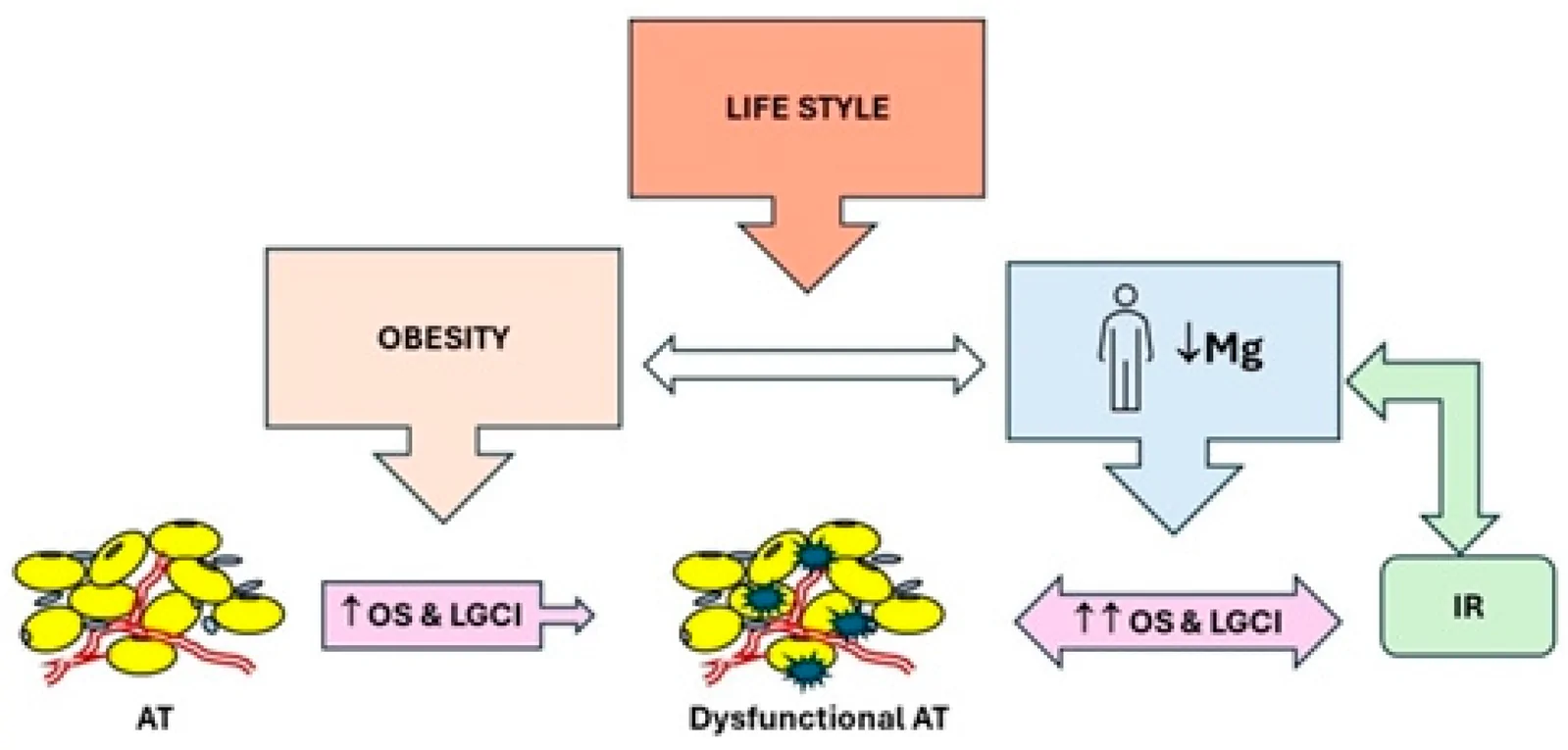
Relationships between Mg and adipose tissue (AT) dysfunction, oxidative stress, inflammation, and insulin resistance in obesity. IR: insulin resistance; LGCI: low-grade chronic inflammation; OS: oxidative stress. ↑: increase; ↓: decrease. Reproduced from Ref. [83] with permission from MDPI.

It has also been reported that Mg deficiency is frequent in obese patients, both in adulthood and in childhood [139]. It should be emphasized that hypomagnesemia in obesity, which is a chronic, relapsing, and multifactorial disease that can increase the risk of serious medical issues, including the prevalence of T2DM [140], can potentiate the excessive production of reactive oxygen species (ROS), mitochondrial dysfunction, and decreased ATP production. More recently, the role of Mg in supporting adipose tissue (AT) metabolism and preventing oxidative stress has been reviewed [141]. This review has highlighted the role of Mg in regulating AT metabolism and its potential to prevent oxidative stress and low-grade chronic inflammation (LGCI) in AT and obesity [141] (Figure 8). This preventive action of Mg is primarily attributed to its role in maintaining mitochondrial function, supporting antioxidant defenses, and acting as a Ca antagonist [142]. It has been suggested that Mg^2+^ deficiency contributes to the development of oxidative stress in obese individuals [143]. In fact, an inadequate intake of Mg has been shown to contribute to the manifestation of its deficiency in obese patients, a condition linked to changes in Ca metabolism and the antioxidant defense system [142]. Conversely, an adequate Mg intake contributes to appropriate homeostasis of this element in the body [143].

## 4. Mechanisms of Hypomagnesemia in Diabetes—A Brief Overview

In the course of T2DM, many pathologies coexisting with this condition may influence magnesium (Mg) homeostasis, leading to its excessive loss and altogether resulting in a vicious-cycle, where a lack of Mg leads to exacerbation of IR, impaired glycemic control, and hyperglycemia, which in turn results in hypermagnesuria [137].

Hypomagnesemia (serum Mg^2+^ concentration ≤ 0.74 mmol/L) is a common coexisting state in patients with T2DM, observed in 13.5–47.7% of the patients [144]. With its complex pathophysiology, it deteriorates the course of the disease in various ways, eventually contributing to the development of disease-related complications [145].

In addition to hypermagnesuria, several mechanisms of hypomagnesemia in T2DM have been proposed, including an inadequate dietary Mg intake [146], the use of some medications (especially metformin or diuretics), and episodes of metabolic acidosis [147,148]. However, this topic still requires more research as well as the issue of the importance and justification of Mg supplementation in diabetic patients.

According to the analysis conducted in the 1970s and 1980s, renal Mg loss was associated with glycosuria [149,150]. With the rising significance of SGLT-2 inhibitors in the treatment of diabetes, this statement was questioned, as these medications reduce glucose reabsorption in the proximal tubule, leading to glucosuria without promoting renal Mg loss [151].

Another postulated mechanism is based on the occurrence of hyperfiltration and increased urinary flow [152]. The Mg concentration gradient is a driving force of reabsorption of this element in the proximal tubule and the thick ascending limb of Henle’s loop. Micropuncture experiments have shown that a tubular fluid-to-interstitial fluid Mg^2+^ concentration ratio of approximately 1.9 is essential for passive reabsorption in the proximal tubule. For this reason, dilution of Mg^2+^ in the forming urine due to hyperfiltration may impair Mg^2+^ reabsorption [153,154].

In diabetic patients, both IR and high blood glucose levels contribute to renal Mg loss, as they impair Mg reabsorption in the thick ascending limb of the loop of Henle, increasing the urinary loss of this element. At the same time, hyperglycemia promotes urinary Mg wasting by increasing osmotic diuresis and changing tubular Mg handling. As blood glucose levels rise, the kidneys become less efficient at reclaiming Mg, resulting in progressively lower serum Mg concentrations [155]. One of the postulated mechanisms of this condition is based on altering transient receptor potential melastatin type 6- and 7-dependent (TRPM6 and TRPM7) Mg transport. Numerous studies have emphasized the role of TRPM6 and TRPM7 in T2DM-associated hypermagnesuria [156]. These ion channels take part in determining intracellular levels of Mg and are regarded as the principal Mg^2+^ entry channels in the distal convoluted tubule of the kidney, where they play a key role in regulating whole-body Mg homeostasis. Insulin modulates the expression of these channels in the plasma membrane through a phosphoinositide 3-kinase (PI3K)–Akt–Rac1 signaling pathway; therefore, any disturbances in insulin action (i.e., IR) may influence TRPM6 activity [157].

Additionally, Mg^2+^ is an essential cofactor for ATP and acts as a key regulator of kinase activity in several intracellular signaling pathways [106], and is an important ion in insulin signaling. Insulin regulates Mg turnover; under physiological conditions, this hormone promotes the movement of Mg^2+^ from the extracellular compartment into cells. IR leads to loss of part of insulin’s ability to stimulate intracellular Mg accumulation resulting in its decrease, reduction in Mg-ATP formation, impairment of insulin receptor tyrosine kinase activity, and weakening of insulin signaling. At the same time, Mg is a crucial factor that determines insulin and glucose homeostasis, playing a pivotal role in carbohydrate metabolism through its impact on tyrosine kinase activity of the insulin receptor and regulation of peripheral insulin sensitivity [158]. Briefly—while insulin binds to its receptor, the receptor undergoes autophosphorylation, leading to the phosphorylation of insulin receptor substrates (IRS-1 and IRS-2). Subsequently, the PI3K/Akt cascade is activated, resulting in glucose transporter 4 (GLUT4) translocation and an increase in glucose uptake [130]. Hypomagnesemia impairs the expression and membrane translocation of GLUT4, leading to diminished glucose transport to insulin-sensitive tissues [159].

Changes in Mg metabolism have been observed not only in diabetes alone but also in many of its complications such as obesity [160], PCOS [161], hypertension [162], dyslipidemia [163], metabolic syndrome [155], diabetic retinopathy and neuropathy, vascular disorders (increased carotid wall thickness, CAD, ischemic stroke), foot ulcerations [147], and inflammation [164].

## 5. Mechanisms of Action of Mg in Diabetes—A Brief Outline

### 5.1. Effect on Glucose Transporter Protein Type-4 (GLUT4)

Mg has been found to mediate effective metabolic control by increasing the level of GLUT4 in diabetic rats [165]. GLUT4 is an insulin-regulated glucose transporter, expressed primarily in adipose tissue and striated muscle, which is a key component in glucose homeostasis and the removal of glucose from circulation [166]. Thus, Mg directly participates in the regulation of glucose translocation into cells.

### 5.2. Effect on Insulin Receptor

Mg may influence the level of the insulin receptor, a member of the ligand-activated receptor and the tyrosine kinase family of transmembrane signaling proteins [167]. It has been reported that the level of insulin receptor, which plays a key role in the regulation of glucose homeostasis [167], was elevated following Mg supplementation in diabetic rats [165].

### 5.3. Role in the Secretion and Action of Insulin

It has been reported that Mg plays a crucial role in the activity of intracellular proteins involved in the secretion of insulin in β-pancreatic cells. It has also been found that this bioelement participates directly in insulin sensitivity [168] and protects against pancreatic cell injury in diabetic rats [169]. Therefore, maintaining adequate Mg levels seems to be crucial for the prevention of metabolic disorders such as diabetes.

### 5.4. Oxidative Stress Reduction

Mg has antioxidant properties that can help reduce oxidative stress caused by an imbalance between the production of ROS in cells and tissues and the ability of a biological system to detoxify them [170], which is closely associated with diabetes [171]. By stimulating antioxidant defense, Mg may protect against free radicals, which are often present in high concentrations in diabetes. Moreover, oxidative stress is increasingly being shown to be a major contributor to the emergence of IR [172], whereas reduction in IR has been found in diabetic rats after Mg supplementation [169].

### 5.5. Anti-Inflammatory Effects

The inflammatory response is one of the main molecular mechanisms underlying the pathophysiology of IR, diabetes, and its related complications [173]. Mg can modulate inflammatory processes, potentially reducing diabetes-associated inflammation, and may thus be a therapeutic option for management of this disease.

### 5.6. Metal Ion Homeostasis

Dysregulation of metal ions, such as copper and iron, i.e., important trace elements for living organisms participating in a range of metabolic processes and homeostatic functions within the human body, is involved in the pathogenesis and progression of diabetes [174,175]. Moreover, electrolyte abnormalities (sodium, potassium, calcium) have been reported to be common in diabetic patients and may be associated with increased morbidity and mortality [176]. Mg can modulate metal ion homeostasis, potentially mitigating the harmful effects of metal ion imbalance. Mg can also interact with vanadium (V) [177], which is considered to be an insulin-mimetic with anti-diabetic effects and is extensively studied as a potential antidiabetic drug [178]. Although the physiological role of this element is unclear, its status as an essential nutrient is under debate, and there are indications that V deficiency may be prejudicial to human health [179]. It has been observed that individuals with normal glucose tolerance (NGT) exhibit mean V blood concentrations of 77.63 ± 19.9 ng/mL (1.4 ± 0.4 μM), which is higher than the concentrations observed in the T2DM population in Portugal, with 59.35 ± 17.7 ng/mL (*p* < 0.001). Thus, the mean V concentrations in NGT individuals have been shown to be 1.3-fold higher than in T2DM patients [179].

### 5.7. Mitochondrial Function

Mitochondrial dysfunction, which takes the central stage in the ever-increasing number of pathologies, is implicated in diabetes [180]. Mg influences mitochondrial functions, which play an essential role in cell survival [181]. It has been reported that the disruption of mitochondrial Mg homeostasis has an unfavorable impact on cellular energy status and cell vulnerability [119]. More information about mitochondria, Mg, and diabetes is provided in the next chapter of this review.

## 6. Mitochondria, Mg^2+^, Oxidative Stress, and Diabetes

Mitochondria are the primary energy producers in cells, generating ATP, the energy currency of the cell. Owing to mitochondria, a healthy adult produces about 50 kg of ATP per day [182]. As described above, after binding with Mg, ATP serves as the native energetic substrate used for various cellular functions [182]. While the primary function of Mg^2+^ is the electrostatic activation of substrates and stabilization of nucleic acids, such as ATP and DNA (Figure 1A,B), respectively, phosphoryl transfer is a fundamental reaction in cellular signaling and metabolism that requires Mg^2+^ as an essential cofactor. Nevertheless, the full spectrum of catalytic mechanisms exerted by Mg^2+^ and the impact of mitochondrial Mg^2+^ alterations on cellular energy metabolism remain unclear [82,119]. The Mg^2+^ concentration, which is remarkably constant and low in the cytosol, is tenfold higher in the mitochondrial matrix. Therefore, mitochondria can be considered the major intracellular Mg^2+^ stores, as described previously [119]. In addition, in mitochondrial respiration, Mg^2+^ mediates the ADP/ATP exchange between the cytosol and the matrix, [MgADP]-dependent mitochondrial ATP synthase activity, and cytosolic free ADP homeostasis [183].

Since mitochondrial dysfunction is strongly associated with diabetes, emerging therapeutic strategies that specifically target mitochondria, such as mitochondria-targeted antioxidants, agents promoting mitochondrial biogenesis, and compounds modulating mitochondrial dynamics, are needed to prevent the development and progression of diabetes, particularly T2DM [184]. T2DM is characterized by mitochondrial dysfunction, a high production of ROS, and low levels of ATP [184]. It has also been observed that aging-related morphological changes in mitochondria may be a direct or indirect consequence of cumulative oxidative damage, which is known to promote cellular and organismal senescence [185]. In diabetes, mitochondria undergo significant morphological changes, often displaying increased fragmentation and reduced size [186]. These alterations can lead to impaired mitochondrial function, including reduced energy production and increased generation of harmful ROS [186].

It has also been described that increased oxidative stress within mitochondria is a common consequence of aberrant mineral homeostasis [187]. It has been proposed that the recommended intake of essential micronutrients should be adjusted in order to ‘tune-up’ metabolism and reduce mitochondrial decay, a hallmark of aging and many disease processes [187]. Eleven of the twelve metals essential for human health have important roles within mitochondrial metabolism. Mg plays a major role in mitochondrial function as well, with one-third or more of the total Mg in a cell located in this organelle [187]. Several studies have associated Mg^2+^ with protection against lipid peroxidation, oxidative stress, and mitochondrial dysfunction [141,142,143,188,189,190]. It has recently been suggested that H_2_O_2_ induces an increase in cytosolic Mg^2+^ concentrations due to dissociation from Mg-ATP, and increased [Mg^2+^]_cyto_-protected mitochondria from ROS-induced damage [188]. Moreover, supplementation of the extracellular medium with Mg^2+^ further suppressed the decrease in MMP (matrix metalloproteinase) and attenuated H_2_O_2_ toxicity. In another study, pretreatment with magnesium sulfate (MgSO_4_) attenuated protein and lipid peroxidation and increased mitochondrial function in mice subjected to different methods of hypoxia [189]. It was further suggested that Mg^2+^ may increase survival time and prevent mortality associated with asphyxiation [189].

Mitochondria have been shown to accumulate Mg ions, especially through Mrs2, an Mg-selective transporter expressed in the mitochondrial inner membrane, which is an essential component of the mitochondrial Mg^2+^ uptake system [119,120,191]. *Mrs2* knockdown in KD cells has been reported to strongly affect intracellular Mg^2+^ levels and energy metabolism. It was suggested that the dysregulation of mitochondrial Mg^2+^ homeostasis disrupted ATP production via a shift in mitochondrial energy metabolism and morphology. It was concluded that mitochondrial Mg^2+^ homeostasis controls cellular energy metabolism and vulnerability to stress [119].

Mg transport by mitochondria depends not only on ATP but also on Ca^2+^ fluxes [192]. The presence of EGTA, which induces a complete absence of extracellular calcium, has been shown to totally prevent mitochondrial Mg^2+^ uptake [192]. When an inhibitor of the Ca^2+^ uniporter was used, Mg^2+^ uptake was blocked. Under these specific experimental conditions, a slow efflux of accumulated Mg^2+^ ions was observed. Clearly, both inward and outward Mg^2+^ movements are affected by Ca^2+^ fluxes across the mitochondrial membrane [192].

## 7. Association of Mg with Metabolism of Other Essential Metals (Cu, Zn, Fe, Mn, Se, and V) in Diabetes

**Figure 9:**
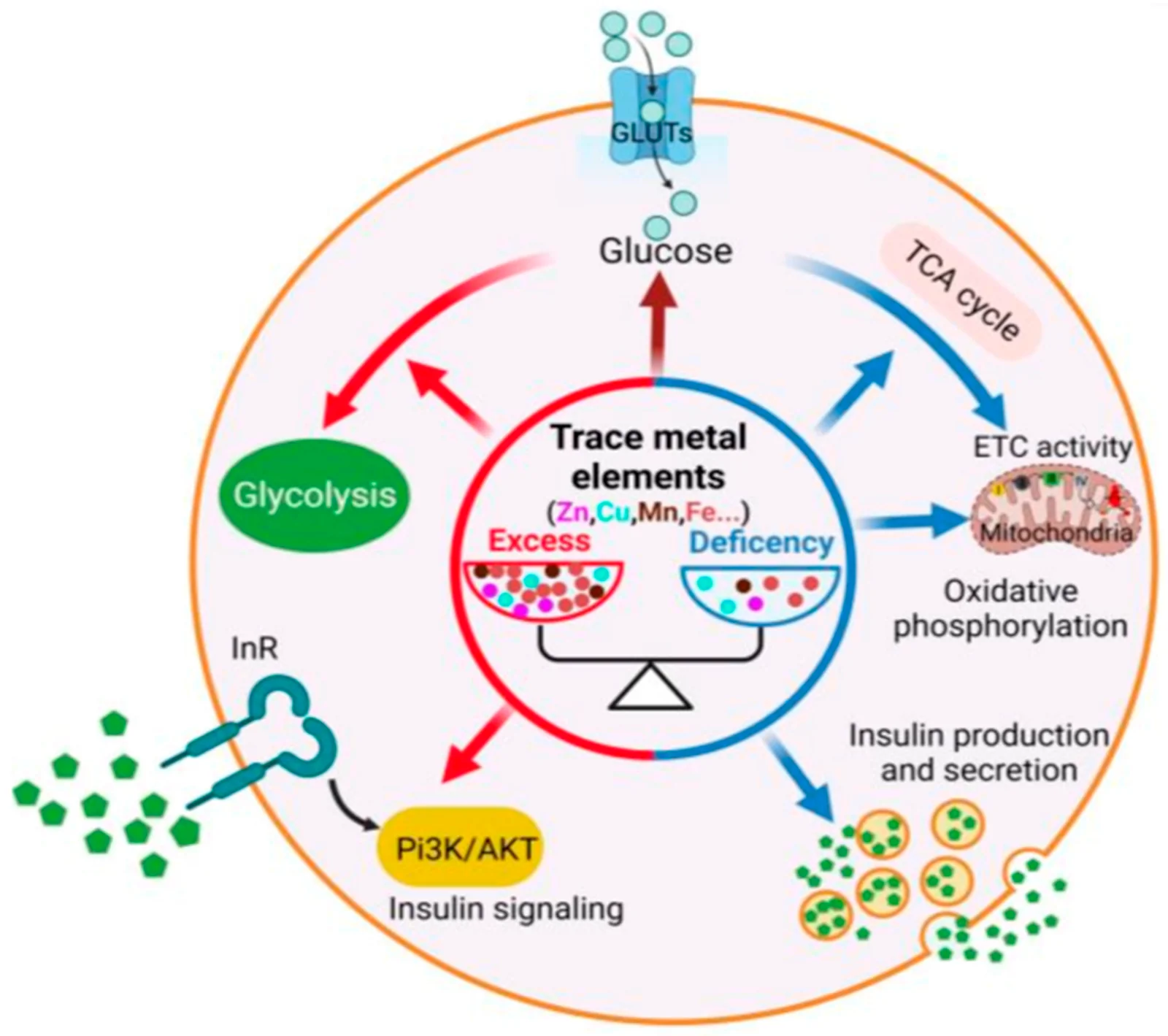

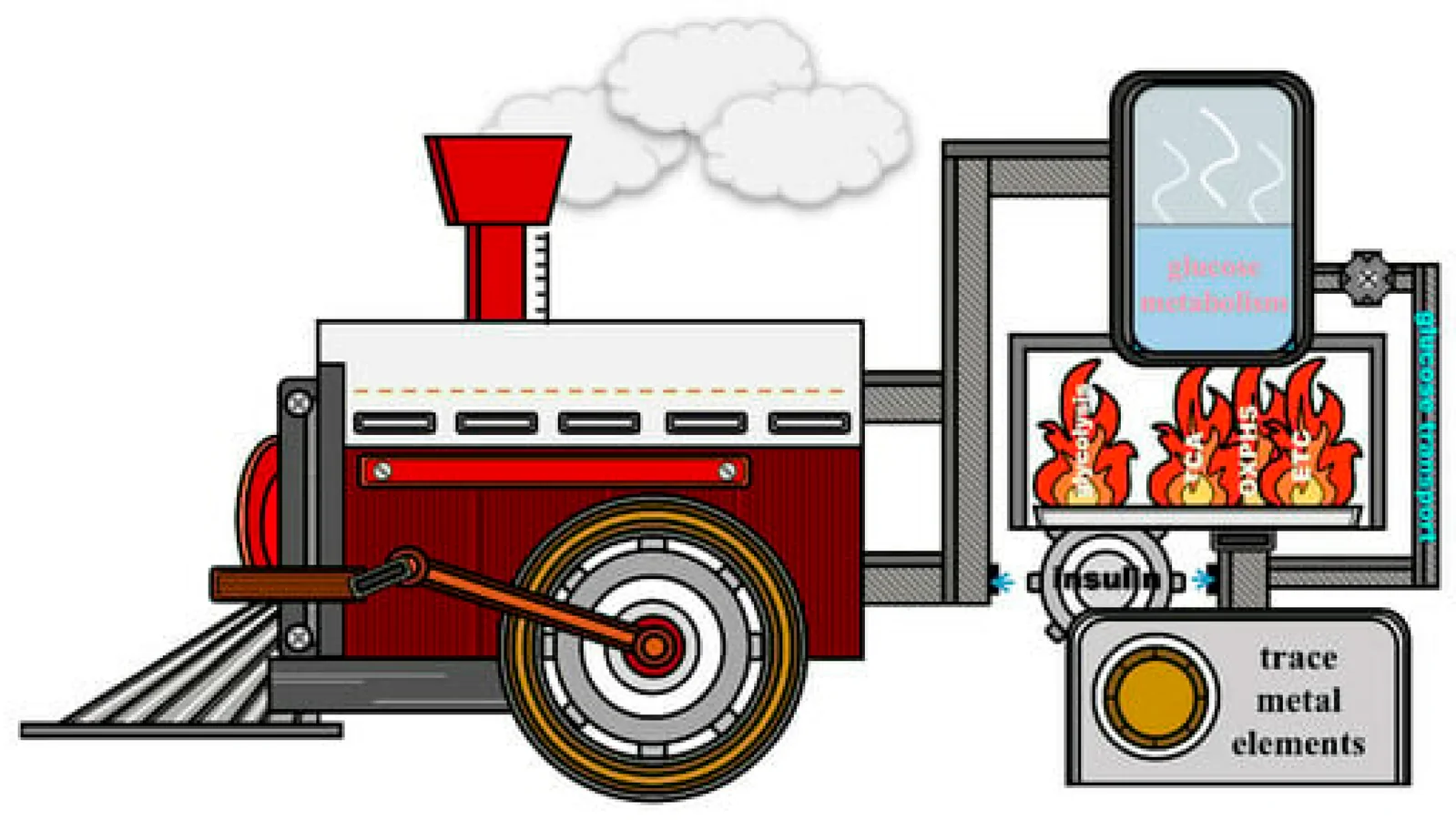
Link between trace metal elements and glucose metabolism: Evidence from zinc (Zn), copper (Cu), iron (Fe), and manganese (Mn)-mediated metabolic regulation. Reproduced from Ref. [83] with permission from MDPI.

Metal ions, including iron (Fe), zinc (Zn), and copper (Cu), play a crucial role in maintaining human health through their balance within the body [193,194]. Disruptions in metal ion balance can intensify diabetic conditions [193]. In diabetes, the metabolism of essential metals like Cu, Zn, and manganese (Mn) is often altered, and these changes can be linked to Mg metabolism (Figure 9). Disruptions in the balance of these metals can affect insulin function and glucose control and may contribute to the development of diabetic complications [193,195]. The interaction between Fe and Mg, which affects the absorption of Fe and its metabolism [196], also plays a significant role in the context of diabetes. Both elements cooperate in glucose metabolism [197,198], and their mutual interaction is crucial for the health of individuals with diabetes. On the other hand, the imbalance of Mg with other metals may lead to the development or aggravation of diabetes. It has been observed that the plasma levels of such elements as Mg, Cu, selenium (Se), and Zn differ between patients with T1DM and T2DM. In T1DM, the plasma Mg concentration undergoes the most pronounced changes and decreases. In T2DM, plasma levels of Se and Cu are affected, and plasma Se and Cu concentrations decrease [195].

A recent study analyzed Mg, Cu, Ca, and Zn in the blood of pregnant women during early pregnancy to evaluate their potential association with gestational diabetes mellitus (GDM). It was observed that higher levels of Fe, Zn, and Mg were positively correlated with GDM risk [199]. In another study, carried out in 2002 in Jiangsu Province (China), a strong inverse association between the Mg:Fe intake ratio and diabetes was found [200]. Based on the obtained findings, the authors stressed that the Mg:Fe intake ratio is an independent risk marker for diabetes in Chinese adults. Also recently, associations between Ca, Mg, Zn, and Cu intakes and the risk of diabetic retinopathy (DR) in US diabetic adults were investigated [201]. It was verified that a higher quartile intake of Ca, Mg, Zn, and Cu was associated with a lower prevalence of DR. It was concluded that higher total Ca, Mg, Zn, and Cu intakes were inversely associated with the risk of DR in US diabetic adults [201]. On the other hand, several studies emphasize the vital protective role of several Mg compounds such as MgSO_4_, Mg (oxide, gluconate, lactate), and Mg aspartate hydrochloride, in the prevention and progression of IR [202]. As V levels are lower in T2DM [179], Mg levels have also been verified to be significantly lower in patients with diabetic retinopathy than in diabetic controls without retinopathy [203]. Besides being accumulated in mitochondria, metals such as V, have been reported to induce beneficial effects in cancerous, diabetic, and neurodegenerative conditions induced by lipid peroxidation and oxidative stress [204].

The present review is an attempt to provide thorough knowledge of the influence of Mg on diabetes in humans. One of the main goals of this work was to provide objective information about a possible beneficial influence of Mg on DM in humans. In other words, we tried to collect data on the efficacy of oral supplementation with Mg on the metabolic profile and the Mg pattern in biological fluids from humans with DM, GDM, IR, and Pre-D. Hence, a reliable analysis of literature data was carried out. Selected findings from studies of glycemic and insulinemic indices as well as the levels of Mg in biological specimens from subjects with T1DM and T2DM, diabetes with serious complications, GDM, IR, or Pre-D, were reviewed and illustrated in an accessible form to readers interested in diabetes and Mg in general.

## 8. Methodology—Literature Search Strategy on Mg and Metabolic Profile in Diabetes in Humans

### 8.1. Databases: General Outline

In this comprehensive review, some English-language databases (i.e., PubMed, Scopus, and Web of Science) were chosen to collect relevant data. Only research articles and abstracts written in English were reviewed. In the case of articles where a full text was unavailable, correspondence was attempted. In the absence of a reply, the information provided in the abstracts was included. Additionally, the reference lists of selected papers collected from the above-mentioned databases were manually reviewed to identify additional records (i.e., full-text papers or abstracts) that were potentially relevant to the topic.

### 8.2. Query Terms Used for the Literature Search on Mg and Metabolic Profile in Diabetes in Humans

The search focused on the ‘Title’ and ‘Abstract’, and such keywords as ‘magnesium’, ‘diabetes mellitus’, ‘supplementation’, ‘blood’, ‘serum’, ‘urine’, and ‘humans’ were used in the search strategy to obtain records on possible changes in the levels of magnesium in biological specimens, i.e., in the blood and urine of patients with diabetes mellitus supplemented with Mg. In addition, such search terms as ‘magnesium’, ‘supplementation’, ‘glycosylated hemoglobin’, ‘fasting plasma glucose’, ‘diabetes’, and ‘insulin resistance’ were also used to obtain records limited to possible changes in the levels of glycemic and insulinemic indices in patients with diabetes, diabetes with serious complications, IR, or prediabetes supplemented with Mg.

### 8.3. Search Results and Literature Review Flowchart on Mg in Diabetes in Humans

**Figure 10:**
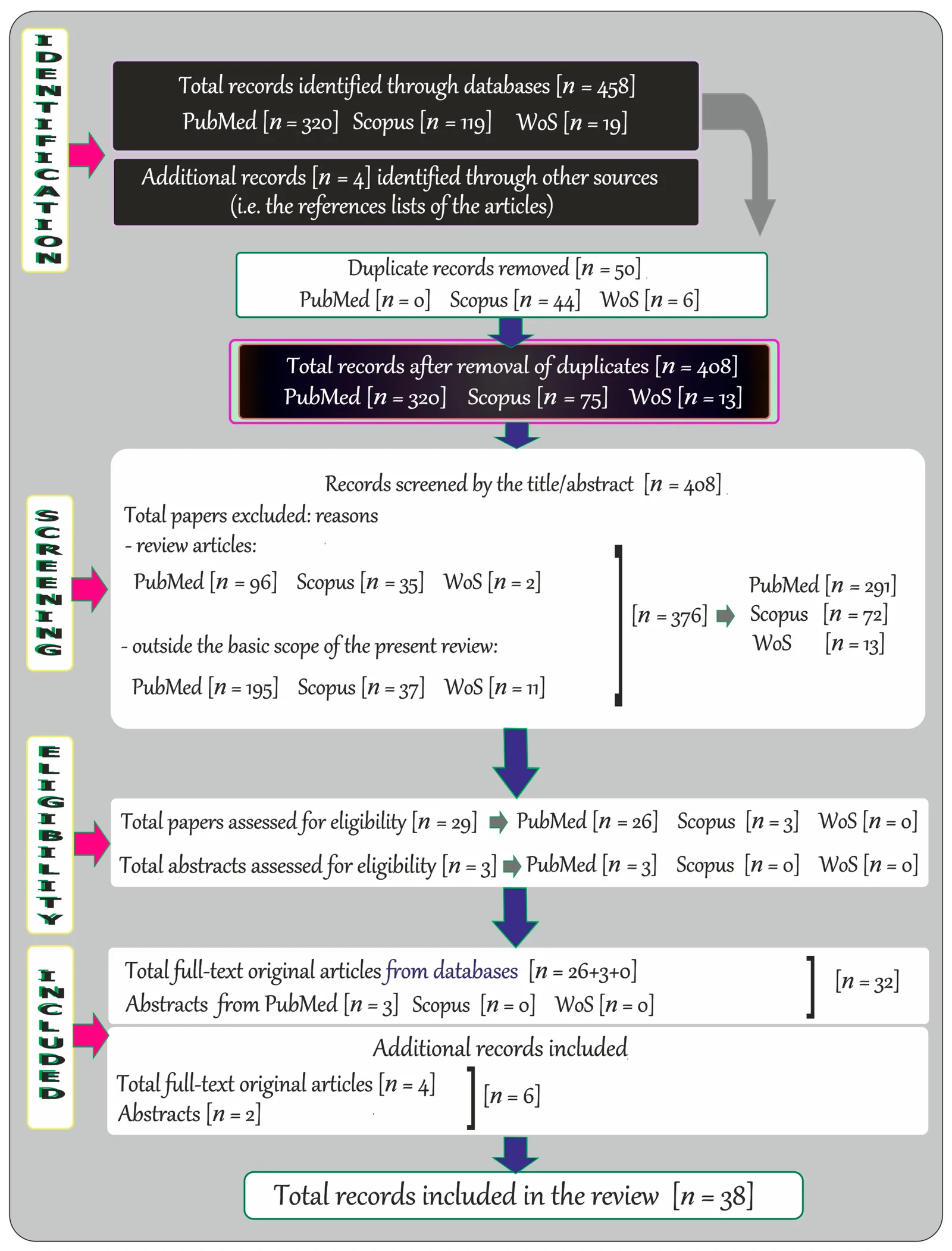
Flow chart of the literature review of studies of the effects of oral Mg supplementation on glycemic and insulinemic indices and the levels of Mg in biological specimens in humans affected by diabetes, diabetes with serious complications, IR, or prediabetes.

The adopted strategy of searching using specific keywords allowed detection of records in PubMed, Scopus, and WoS that were relevant to the topic of Mg and diabetes in humans. The flow chart provided below (Figure 10) shows the process employed to identify records of the effects of oral Mg supplementation on glycemic and insulinemic parameters as well as the concentration of Mg in biological specimens in humans with diabetes, diabetes with serious complications, IR, or Pre-D.

An extensive search of major electronic databases (PubMed, Scopus, and WoS) was conducted from January 2026 to March 2026 to identify relevant studies published on the effects of oral Mg supplementation on glycemic and insulinemic indices and on the levels of Mg in biological specimens in humans affected by diabetes, diabetes with serious complications, IR, or prediabetes. A total of 458 records published in English were identified through the databases listed above; i.e., 320 through PubMed, 119 through Scopus, and 19 through WoS. The majority of the articles that were relevant to the topic were selected by the first author of this review. After the initial research, articles that were relevant to the topic were selected by applying the following steps: removal of duplicate articles, removal of review papers, removal of articles that were outside the basic scope of this review, and evaluation of titles/abstracts to obtain articles that were potentially relevant. More precisely, after removal of 50 duplicate items (PubMed: 0, Scopus: 44, and WoS: 6), the remaining records (n = 408; PubMed: 320, Scopus: 75, and WoS: 13) were initially screened by title and abstracts. Afterwards, review articles and those that did not address the topic were excluded (PubMed: 291, Scopus: 72, and WoS: 13). Next, a total of 29 potentially relevant full-text articles (PubMed: 26 and Scopus: 3) and three abstracts (PubMed) were further examined. Moreover, six additional records (i.e., four full-text original articles and two abstracts) relevant to the topic were included by manual review of bibliographies. Finally, a total of 38 records (i.e., 29 full text original papers and five abstracts) were included in the current review.

## 9. Effects of Oral Mg Supplementation on Diabetes: Humans—A Summarizing Note

**Figure 11:**
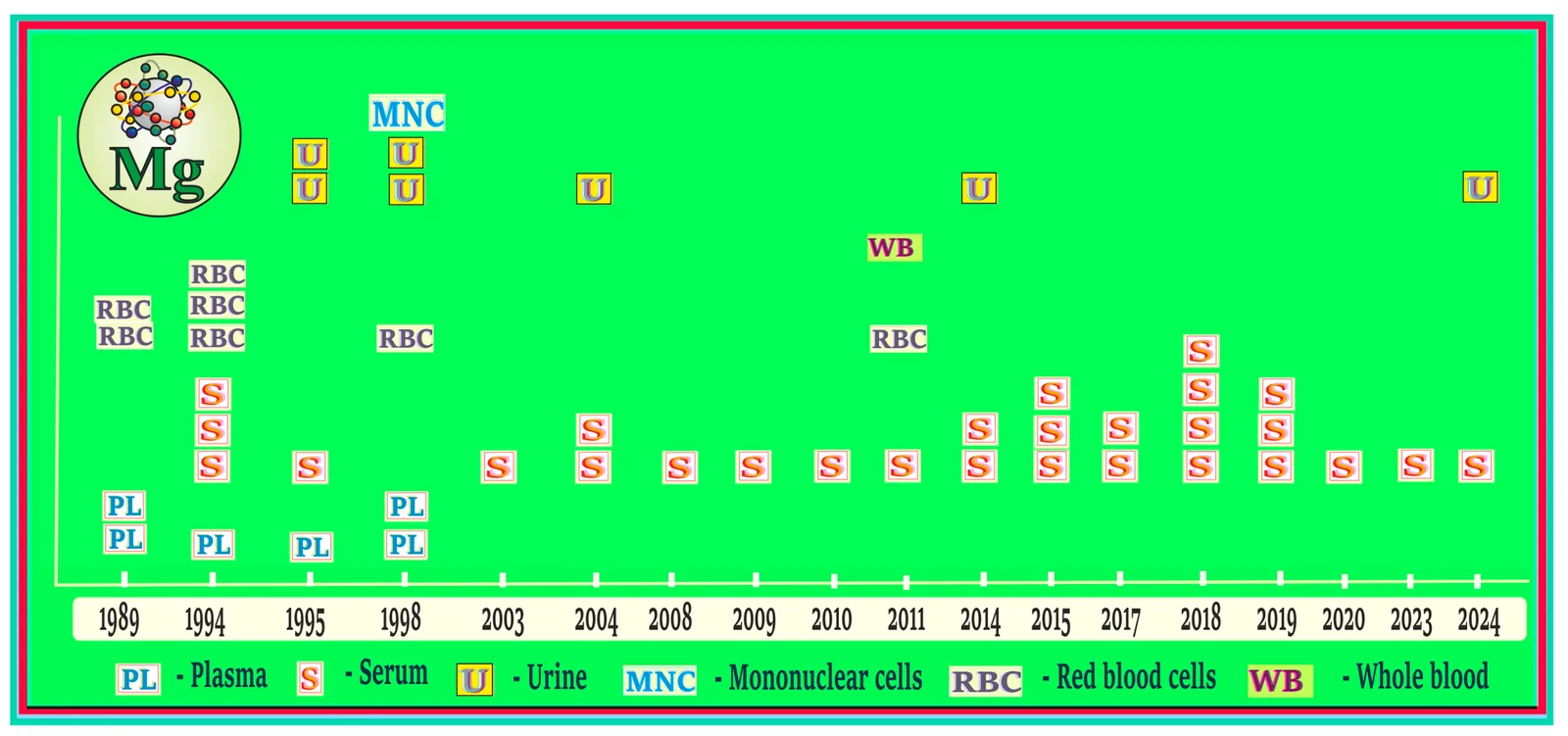
Summary of studies on the determination of the level of Mg in plasma (PL), serum (S), mononuclear cells (MNS), erythrocytes (RBS), whole blood (WB), and urine (U) in individuals with diabetes mellitus, diabetes with serious complications, gestational diabetes, IR, or prediabetes on the timeline—based on the available literature provided in Table 2.

Since Mg is associated with INS homeostasis and glucose metabolism and since hypomagnesemia, which is defined as a serum Mg concentration ≤ 1.8 mg/dL (≤0.74 mmol/L) [205], has been reported to be linked to diabetes [137] and diabetic complications [206], we summarized the results of studies on the efficacy of oral Mg supplementation in humans with hypo- and normomagnesemia suffering from T1DM and T2DM, diabetes with serious complications, GDM, IR, or Pre-D with respect to the metabolic profile (Table 1). Additionally, we summarized the results of studies on the levels of Mg in biological specimens, i.e., plasma (PL), serum (S), mononuclear cells (MNS), erythrocytes (RBS), whole blood (WB), and urine (U), in subjects with hypo- and normomagnesemia suffering from diabetes, diabetes with serious complications, GDM, IR, or Pre-D after oral Mg supplementation. Details concerning these studies are presented in Figure 11 and Table 1 and Table 2.

As shown, among the above-mentioned biological samples, S was used most often for determination of the concentration of Mg, followed by RBC, PL, and U. In turn, MNC and WB were the least frequently tested samples.

The first reports (two papers) on the detection of Mg in biological samples (i.e., PL and RBC) obtained from patients with T2DM were issued at the end of the 20th century, i.e., in 1989. The subsequent articles specifying the levels of this mineral in the PL, S, and RBC of T2DM patients appeared in the 1990s, i.e., in 1994. The next two papers presenting the concentration of Mg in the S/PL and U of T2DM/T1DM subjects were published in 1995. Further, two articles on the level of Mg in the PL, RBC, MNC, and U of T2DM patients appeared in 1998. At the beginning of the 21st century, three papers (one in 2003 and two in 2004) reported concentrations of Mg in the S and/or U. Subsequent papers were published in 2008, 2009, 2010, 2011, 2014, 2015, 2017, 2018, 2019, 2020, 2023, and 2024.

A clinical randomized, double-blinded, placebo-controlled trial on the impact of oral supplementation with Mg on glycemic parameters in subjects with hypomagnesemia and the most common form of DM, i.e., T2DM, was performed by De Lourdes Lima et al. [207]. The authors did not find any significant changes in fasting plasma glucose (FPG), which is the gold standard diagnostic criterion for T2DM [243], or in glycosylated hemoglobin (HbA1c), which is considered a useful marker for evaluating glycemic control in diabetes [244], after treatment with Mg supplemented as Mg oxide (MgO, 41.4 mmoL/day) for 30 days. In this study, patients who received 41.4 mmoL of MgO daily exhibited a tendency toward an increase in the plasma Mg level and a significant increase in urinary Mg excretion (Table 1 and Table 2). No statistically significant changes in FPG and/or HbA1c accompanied by elevated serum Mg levels in Type 2 diabetic and hypomagnesemic patients were found by Guerrero-Romero and Rodriguez-Moran [208] and Eibl et al. [210], who performed randomized, double-blinded, placebo-controlled trials, after oral supplementation with Mg chloride (MgCl_2_, 450 mg/d of elemental Mg) for 4 months and Mg citrate (30 mmoL/d, 730 mg/d of elemental Mg) for 3 months, respectively (Table 1 and Table 2). Similarly, no statistically significant changes in FPG and/or HbA1c accompanied by elevated serum Mg levels in Type 2 diabetic and hypomagnesemic patients were demonstrated by Barragan-Rodriguez et al. [209], Drenthen et al. [211], or Halawa et al. [212] after oral Mg supplementation using Mg chloride (MgCl_2_, 450 mg/d of elemental Mg) for 12 weeks, Mg gluconate (15 mmoL/d, 360 mg/d) for 6 weeks, and Mg citrate (15 mmoL, 360 mg/d of elemental Mg) for 12 weeks, respectively (Table 1 and Table 2). Barragan-Rodriguez et al. [209], Drenthen et al. [211], and Halawa et al. [212] performed a randomized controlled trial; a randomized, double-blinded placebo-controlled, cross-over study; and a prospective, randomized, controlled, open-label trial, respectively. Based on these findings, it can be concluded that oral Mg supplementation corrects hypomagnesemia in Type 2 diabetic subjects but does not affect glycemic control. The lack of a significant effect of oral Mg supplementation on glycemic indices, despite an increased Mg level in blood and, in some cases, also in urine, may be attributed inter alia to the short duration of supplementation with this mineral. Moreover, in studies performed by Guerrero-Romero and Rodriguez-Moran [208], Barragan-Rodriguez et al. [209], and Halawa et al. [212], hypomagnesemic patients with T2DM were also diagnosed with hypertension, depression, and diabetic nephropathy, respectively. In these cases, oral Mg supplementation reduced blood pressure [208], exhibited effectiveness in the management of depression in the elderly [209], and improved microalbuminuria [212], which is considered a predictor of worse renal outcomes [245].

Rodríguez-Morán and Guerrero-Romero [216] conducted a randomized, double-blinded, placebo-controlled clinical trial to determine whether oral Mg supplementation improves glycemic control and INS sensitivity in Type 2 diabetic subjects with low serum Mg levels. They noted reductions in FPG, glycosylated hemoglobin (HbA1c), and the IR index (HOMA-IR)— which is used for measuring INS sensitivity [246]— and a higher serum Mg level in hypomagnesemic diabetic subjects supplemented with MgCl_2_ (2.5 g/d) for 16 weeks. Similar findings, i.e., a decrease in levels of FPG and HbA1c and a decline in the HOMA-IR index, were reported by Afazi et al. [217], who performed a randomized, double-blinded, placebo-controlled trial to determine the effects of Mg and vitamin E (vit. E) co-supplementation (Mg oxide + vit. E, 250 mg/d + 400 IU/d, 12 weeks) on wound healing and metabolic status in hypomagnesemic patients with diabetic foot ulcer (DFU). A decrease in HbA1c level in hypomagnesemic children with T1DM receiving MgO (300 mg) for 3 months and a decrease in FPG levels in individuals with T2DM after oral supplementation of MgCl_2_ (300 mg) for 4 months were demonstrated by Shahbah et al. [218] and Singh et al. [219], respectively. In both cases, an increase in serum Mg levels was noted (Table 1 and Table 2). Furthermore, in a randomized, double-blind, placebo-controlled trial aimed at determining whether oral Mg supplementation in the form of Mg chloride (MgCl_2_, 300 mg/d) for 3 months can modify INS sensitivity in non-diabetic IR subjects with hypomagnesemia, Guerrero-Romero et al. [214] reported a reduced FPG level and a lower HOMA-IR index in individuals receiving this bioelement. Guerrero-Romero’s research team [215] also reported a decrease in FPG level and the HOMA-IR index in subjects with pre-diabetes with hypomagnesemia after supplementation with MgCl_2_ (382 mg of Mg) for 4 months. In both studies, the authors demonstrated that these changes were accompanied by elevated serum Mg levels (Table 1 and Table 2). Based on these findings, it can be concluded that oral Mg supplementation improves glycemic status and/or INS sensitivity in hypomagnesemic Type 2 diabetic subjects, in hypomagnesemic patients with DFU, in hypomagnesemic children with T1DM, in hypomagnesemic non-diabetic subjects with IR, and in hypomagnesemic individuals with pre-diabetes. 

In normomagnesemic subjects suffering from diabetes, no beneficial effect of oral Mg supplementation administered as Mg aspartate (15 mmoL/d), Mg pidolate (16.2 mmoL/d, 15.8 mmoL/d, 368 mg/d), Mg lactate-citrate (15 mmoL/d), or Mg chloride (1.9 mmoL/d) for 3, 1, 4, and 1.5 months, respectively, on the FPG and/or HbA1c levels in Type 2 diabetic individuals was found by De Valk et al. [220], Corica et al. [222], Paolisso et al. [225], Barbagallo et al. [226] Gullestad et al. [223], and Purvis et al. [227]. These researchers performed a randomized, double-blinded, placebo-controlled trial; a randomized, placebo-controlled trial; a randomized, double-blinded, cross-over study; a controlled trial; a randomized, double-blinded, placebo-controlled trial; and a randomized, double-blinded, placebo-controlled, cross-over study, respectively. Navarrete-Cortes et al. [224], who performed a randomized, double-blinded, placebo-controlled, cross-over study, did not demonstrate any significant changes in the FPG and HbA1c levels, and additionally in HOMA-IR in Type 2 diabetic subjects supplemented with Mg lactate (360 mg/d of elemental Mg) for 3 months. Similarly, Eriksson and Kohvakka [221], who performed a randomized, double-blinded, placebo-controlled study, reported no significant alterations in the FPG and HbA1c levels in Type 2 diabetic patients after a three-month treatment with Mg (unspecified Mg compound, 24.7 mmoL/d) (Table 1 and Table 2). The absence of significant differences in these indices was noted despite elevated or unchanged serum/plasma Mg levels during Mg supplementation. These findings suggest that oral Mg supplementation at the doses and duration studied did not have an impact on diabetes-related parameters in normomagnesemic subjects with T2DM.

In turn, in studies conducted by other authors, who performed a randomized, double-blinded, placebo controlled trial and a randomized, cross-sectional, controlled study, glycemic and INS sensitivity indices in diabetic normomagnesemic individuals exhibited a different pattern of changes. For example, Solati et al. [228] reported an unchanged HOMA-IR index and HbA1c level but a reduced FPG level that was accompanied by an unaltered serum Mg level and elevated urinary Mg excretion. Yokota et al. [229] showed a decreased HOMA-IR index and unaltered HbA1c and FPG levels together with an elevated serum Mg concentration and urinary Mg excretion. Sadeghian et al. [231] noted an elevated HOMA-IR index and unchanged HbA1c and FPG levels with an unchanged serum Mg level (unspecified urine Mg concentration), and Albaker et al. [230] reported a reduced HOMA-IR index and HBA1c level but unchanged FPG (unspecified serum/urine Mg concentration) in normomagnesemic Type 2 diabetic individuals administered with different Mg compounds (Mg sulfate, Mg chloride, and Mg oxide) at different doses (300 mg/d, 250 mg/d, 50 mg/L) for varying durations, i.e., 30 days or 3 months (Table 1 and Table 2). The differences in the above-mentioned results can be explained by the differences in the doses of Mg and the duration of supplementation with this bioelement. Taking into account the results indicating a significant decrease in the HOMA-IR index [229,230], FPG level [228], and HbA1c level [230], it can be concluded that oral Mg supplementation in normomagnesemic subjects with diabetes (T2DM) can be helpful in the control of this disease.

A decreased HOMA-IR index and HbA1c and/or FPG in normomagnesemic subjects with T2DM, GDM, or IR with an increased plasma/serum Mg level after oral supplementation with different Mg compounds (i.e., Mg tablets: oxide, gluconate, lactate, Mg oxide, Mg pidolate, Mg aspartate hydrochloride) alone or in combination with, e.g., Vitamin E., zinc sulfate, or zinc–calcium–vitamin D and different doses (250 mg/d, 2 g/d, 365 mg/d, 100 mg) and after different durations of administration (4, 6, 12, 24 weeks) were demonstrated by other researchers who performed randomized controlled trial; randomized, double-blinded, placebo-controlled trials; and a randomized cross-over study [232,233,234,235,236,237,238,239,240] (Table 1 and Table 2). The results obtained by these authors indicate that oral Mg supplementation improves glycemic and/or insulinemic indicators in normomagnesemic subjects with T2DM, GDM, or IR.

It should be emphasized that every supplementation should be carried out with caution bearing in mind the safety profile as both element deficiency and excess may be dangerous. Mg supplements are widely used for potential benefits including hypomagnesemia, muscle relaxation and sleep improvement [247]. Common forms found in the supplements are magnesium oxide, citrate, chloride, aspartate, gluconate, and oronate, of which organic Mg salts exhibit higher bioavailability. Concomitant administration with food may improve gastrointestinal tolerability and optimize absorption [248,249].

Oral Mg supplementation is generally well tolerated at recommended doses. According to Costello et al. [250], one-third of Americans consume a Mg supplement with a mean dose of 146 mg/day. In the U.S., the upper limit for this element in dietary supplements and medications is 350 mg/day; however, it does not include Mg that is naturally present in the diet and is largely determined by the occurrence of gastrointestinal adverse effects, particularly diarrhea [251]. The RDA (Recommended Daily Allowance) ranges from 410 to 420 mg/day for adult men and 310–400 mg/day for adult women (including pregnant and lactating women) [252,253]. According to the Dietary Reference Values for the Polish population, the recommended total daily Mg intake is similar: 400–420 mg/day for adult men and 310–400 mg/day for adult women (including pregnant and lactating women) [254]. These thresholds refer to the total Mg intake and should not be interpreted as recommended doses of Mg supplementation, as there are no recommendations establishing a single universal supplemental Mg dose for all healthy adults.

When it comes to specific values, serum Mg concentrations ranging from 1.05 to 2.2 mmol/L (2.55–5.35 mg/dL) are generally well tolerated and may remain asymptomatic. At concentrations of 2.2 and 3.5 mmol/L (5.35–8.5 mg/dL) patients may experience dizziness, cutaneous flushing, and weakness. Values above 3.5 mmol/L (8.5 mg/dL) are linked to neuromuscular and neurological symptoms such as a reduction in deep tendon reflexes, bladder paralysis, headache, confusion, and hypotension [115,124]. Concentrations above 6.5 mmol/L (15.8 mg/dL) lead to life threatening conditions of which gastrointestinal atony, neuromuscular paralysis, respiratory depression, coma and cardiac arrest are the most serious ones [115,255].

## 10. Summary and Conclusions

In conclusion, some of the findings compiled in the present review report beneficial effects of Mg intervention in patients with hypo- or normomagnesemia and diabetes or IR. On the other hand, certain studies did not show any significant effects of supplementation with this mineral on the improvement of glycemic and/or insulinemic parameters in hypo- or normomagnesemic subjects with diabetes or who are at risk of the disease. Simultaneously, it should be emphasized that the included studies feature substantial heterogeneity in magnesium formulations (i.e., oxide, chloride, citrate, pidolate, and sulfate with distinct bioavailability), elemental magnesium dosages, intervention duration, baseline patient profiles, and concurrent medications. Therefore, based on the findings presented in the current review, it can be concluded that further long-term studies using effective Mg dosages and a larger number of participants seem to be necessary so as to definitively establish its benefits in improving diabetes outcomes or preventing the development of serious complications. Moreover, the introduction of a routine determination of blood Mg levels—especially the Mg-ionized form, as the total serum Mg concentration does not reflect the systemic Mg level—could be used in diabetic patients, as well as in those at risk of IR and glucose metabolism disorders. However, the clinical utility of routine magnesium monitoring requires further investigation. Additionally, it remains to be clearly determined which patients could benefit most from supplementation with Mg in the context of diabetes. It should be added that some populations may be particularly predisposed to develop more severe adverse reactions and hypermagnesemia. These are elderly people due to impaired renal function and a higher possibility of drug interactions, pregnant women; patients with chronic kidney disease; or patients with any disturbances in renal excretion while simultaneously receiving Mg containing medications [252]. Iatrogenic hypermagnesemia occurs in patients receiving laxatives (magnesium hydroxide) [256], antacids [257] or intravenous Mg infusions (magnesium sulfate used in eclampsia treatment) [258]. These patients require regular serum Mg levels monitoring. In healthy adults with intact renal function the risk of overdose is low [259]. Moreover, oral Mg supplements may interact with concomitant medication and influence therapy effectiveness. Depending on the mechanism, drug–drug- interactions may be based on chelation and complex formation (antibiotics—mainly fluoroquinolones and tetracyclines [260,261]; bisphosphonates [261]; or integrase inhibitors used in HIV treatment [262]) or increased drug absorption (nonsteroidal anti-inflammatory drugs, sulfonylurea antidiabetic agents or oral anticoagulants [263]). Proton-pump inhibitors and diuretics on the other hand may decrease one’s Mg status [264].

Thus, whether supplementation with Mg—which has antioxidant and anti-inflammatory properties, supports mitochondrial function, and thus plays a crucial role in the secretion and action of insulin as well as metal ion homeostasis—should be considered an integral part of diabetes management remains an open question.

## Figures and Tables

**Table 1:** Summary of the results on studies of the effects of oral Mg supplementation on the metabolic profile in humans with diabetes, diabetes with serious complications, IR, or prediabetes.

Type of Study	Condition	Number of Treated Subjects	Age (Years, Mean ± SD)	Mg Compound/Dosage	Time of Treatment	Results	Ref.
Randomized, double-blinded, placebo-controlled	T2DM Hypo-Mg	n = 35	55.4 ± 10.2	Mg oxide 20.7 mmoL/d	30 d	FPG → HbA1c →	[207]
Randomized, double-blinded, placebo-controlled	T2DM Hypo-Mg	n = 39	51.2 ± 11.0	Mg oxide 41.4 mmoL/d	30 d	FPG → HbA1c →	[207]
Randomized, double-blinded, placebo-controlled	T2DM and HBP Hypo-Mg	n = 40	58.9 ± 8.5	Mg chloride (450 mg/d of elemental Mg)	4 mo	FPG → HbA1c →	[208]
Randomized controlled clinical trial	T2DM and depression Hypo-Mg	n = 12	69 ± 5.9	Mg chloride (450 mg/d of elemental Mg)	12 wk	FPG → HbA1c →	[209]
Randomized, double-blinded, placebo-controlled	T2DM Hypo-Mg	n = 18	63 ± 8.0	Mg citrate 30 mmoL/d (730 mg/d of elemental Mg)	3 mo	HbA1c →	[210]
Randomized, double-blinded, placebo-controlled, cross-over	T2DM Hypo-Mg	n = 14	67 ± 6.0	Mg gluconate 15 mmoL/d (360 mg/d of elemental Mg)	6 wk	HbA1c →	[211]
Prospective, randomized, controlled, open-label	Type 2 DN Hypo-Mg Normo-Mg	n = 26 n = 14 n = 12	61.4 ± 7.5	Mg citrate 15 mmoL (360 mg/d of elemental Mg)	12 wk	HbA1c → HbA1c →	[212]
N/A	DM Hypo-Mg	n = 40	N/A	Mg oxide 600 mg	12 wk	FPG →	[213]
Randomized, double-blinded, placebo-controlled	IR Hypo-Mg	n = 32	43 ± 7.9	Mg chloride 2.5 g (300 mg/d of elemental Mg)	3 mo	↓ HOMA-IR ↓ FPG	[214]
Randomized, double-blinded, placebo-controlled	Pre-D Hypo-Mg	n = 59	42.5 ± 9.5	Mg chloride (382 mg/d of elemental Mg)	4 mo	↓ HOMA-IR ↓ FPG	[215]
Randomized, double-blinded, placebo-controlled	T2DM Hypo-Mg	n = 32	59.7 ± 8.3	Mg chloride 2.5 g/d (12.8 mmoL/d)	16 wk	↓ HOMA-IR ↓ FPG ↓ HbA1c	[216]
Randomized, double-blinded, placebo-controlled	Grade 3 DFU Hypo-Mg	n = 29	57.2 ± 11.0	Mg oxide + Vit. E 250 mg/d + 400 IU/d	12 wk	↓ HOMA-IR ↓ FPG ↓ HbA1c	[217]
Observational	T1DM Hypo-Mg	n = 20	11.2 ± 3.41	Mg oxide 300 mg	3 mo	↓ HbA1c	[218]
Controlled	T2DM Hypo-Mg	n = 60	N/A	Mg chloride tablet 300 mg/d	16 wk	↓ FPG	[219]
Randomized, double-blinded, placebo-controlled	T2DM	n = 25	63 ± 8.2	Mg aspartate 15 mmoL/d	3 mo	FPG → HbA1c →	[220]
Randomized, double-blinded, cross-over	T2DM	n = 27	61 ± 2.0	Mg (unspecified compound) 600 mg/d (24.7 mmoL/d)	90 d	FPG → HbA1c →	[221]
Randomized, double-blinded, cross-over	T1DM	n = 29	43 ± 2.0	Mg (unspecified compound) 600 mg/d (24.7 mmoL/d)	90 d	FPG → HbA1c →	[221]
Randomized, placebo-controlled	T2DM	n = 26	63 ± 5.0	Mg pidolate 4.5 g/d (16.2 mmoL/d)	1 mo	FPG → HbA1c →	[222]
Randomized, double-blinded, placebo-controlled	T2DM	n = 25	64 ± 8.0	Mg lactate-citrate 15 mmoL/d (184.5 mg/d of elemental Mg)	4 mo	FPG → HbA1c →	[223]
Randomized, double-blinded, placebo-controlled, cross-over	T2DM	n = 56	52.84 ± 8.42	Mg lactate 1.5 g/d (360 mg/d of elemental Mg)	3 mo	FPG → HbA1c→ HOMA-IR →	[224]
Randomized, double-blinded, cross-over	T2DM	n = 4	73 ± 2.5	Mg pidolate 4.5 g/d (15.8 mmoL/d)	4 wk	FPG → Improvement of INS sensitivity and GLU oxidation	[225]
Controlled	T2DM	n = 30	71.1 ± 6.1	Mg pidolate 368 mg/d	1 mo	FPG →	[226]
Randomized, double-blinded, placebo-controlled, cross-over	T2DM	n = 28	28–84	Mg chloride 384 mg/d (1.9 mmoL/d)	6 wk	GLU →	[227]
Randomized, double-blinded, placebo-controlled	T2DM	n = 25	46.76 ± 6.93	Mg sulfate 300 mg/d of elemental Mg	3 mo	HOMA-IR →, ↓ FPG HbA1c →	[228]
N/A	T2DM	n = 9	51.6 ± 2.6	MAG21 solution (Mg chloride), 300 mg/d	30 d	↓ HOMA-IR, FPG → HbA1c →	[229]
Randomized, cross-sectional, controlled clinical trial	T2DM	n = 32	55.9 ± 8.9	Mg chloride 50 mg/L daily	3 mo	↓ HOMA-IR, FPG → ↓ HbA1c	[230]
Randomized, double-blinded, placebo-controlled	Type 2 DN	n = 40	41.2 ± 8.8	Mg oxide 250 mg (150 mg of elemental Mg)	12 wk	↑ HOMA-IR, FPG → HbA1c →	[231]
Randomized controlled clinical trial	T2DM	n = 20	35–60	Mg tablets (oxide, gluconate, lactate) 250 mg/d of elemental Mg	3 mo	↓ HOMA-IR, ↙ FBS ↓ HbA1c	[232]
Randomized, double-blinded, placebo-controlled	T2DM with grade 3 DFU	n = 35	60.1 ± 11.1	Mg oxide 250 mg/d	12 wk	↙ HOMA-IR, ↓ FPG ↓ HbA1c	[233]
Randomized, double-blinded, placebo-controlled	DHD	n = 27	58.8 ± 10.1	Mg oxide 250 mg/d	24 wk	↓ HOMA-IR ↙ FPG ↓ HbA1c	[234]
Randomized, double-blinded, placebo-controlled	GDM	n = 30	30.1 ± 5.9	Mg oxide + Vit. E 250 mg/d + 400 IU/d	6 wk	↓ HOMA-IR, ↓ FPG	[235]
Randomized, double-blinded, placebo-controlled	T2DM and CHD	n = 27	61.7 ± 9.4	Mg oxide + Zn sulfate 250 mg/d + 150 mg/d	12 wk	↓ FPG	[236]
Randomized, cross-over	T2DM	n = 8	72.2 ± 2.0	Mg pidolate 2 g/d (7.0 mmoL/d)	4 wk	↓ FPG Improvement of INS response and action	[237]
Randomized, double-blinded, placebo-controlled	GDM	n = 20	27.8 ± 3.4	Mg oxide 250 mg/d	6 wk	↓ FPG	[238]
Double-blinded, placebo-controlled	GDM	n = 30	N/A	Mg + Vit. E 250 mg/d + 400 mg/d	6 wk	↓ FPG Improvement of INS sensitivity	[239]
Randomized, double-blinded, placebo-controlled	IR	n = 25	30–70	Mg aspartate hydrochloride 15 mmoL (365 mg/d of elemental Mg)	6 mo	↓ FPG ↓ ISI-HOMA	[240]
Randomized, double-blinded, placebo-controlled	T1DM	n = 3	N/A	Mg hydroxide 20–30 mmoL/d	12 mo	↑ HbA1c	[241]

Ca: calcium; CHD: coronary heart disease; d: day; DM: diabetes mellitus; DFU: diabetic foot ulcer; DHD: diabetic hemodialysis patients; DN: diabetic nephropathy; FBS: fasting blood sugar; FPG: fasting plasma glucose; GDM: gestational diabetes; GLU: glucose; HbA1c: glycosylated hemoglobin; HOMA-IR: homeostasis model assessment as an index of insulin resistance; HBP: hypertension; Hyp-Mg: hypomagnesemia; IR: insulin resistance; ISI: insulin sensitivity indices; Mg: magnesium; mo: month; Normo-Mg: normomagnesemia; Pre-D: prediabetes; T2DM: Type 2 diabetes mellitus; Vit. D: vitamin D; Vit. E: vitamin E; wk: weeks; Zn: zinc. N/A: not available. ↑: increase; ↓: decrease; ↙: trend towards a decrease; → without changes.

**Table 2:** Summary of the levels of Mg in biological specimens from humans with diabetes, diabetes with serious complications, gestational diabetes, IR, or prediabetes after oral Mg supplementation.

Type of Study	Condition	Number of Treated Subjects	Age (Years, Mean ± SD)	Mg concentration After Treatment	Biological Specimens	Units	Ref.
Randomized, double-blinded, placebo-controlled	T2DM Hypo-Mg	n = 35	55.4 ± 10.2	0.76 → 1.59 → 121 ↑	Plasma MonoNC Urine	mmol/L μg/mg TP mg/24 h	[207]
Randomized, double-blinded, placebo-controlled	T2DM Hypo-Mg	n = 39	51.2 ± 11.0	0.80 ↗ 1.62 ↗ 113 ↑	Plasma MonoNC Urine	mmol/L μg/mg TP mg/24 h	[207]
Randomized, double-blinded, placebo-controlled	T2DM and HBP Hypo-Mg	n = 40	58.9 ± 8.5	0.81 ↑	Serum	mmol/L	[208]
Randomized controlled clinical trial	T2DM and depression Hypo-Mg	n = 12	69 ± 5.9	0.86 ↑	Serum	mmol/L	[209]
Randomized, double-blinded, placebo-controlled	T2DM Hypo-Mg	n = 18	63 ± 8.0	0.81 ↑ 2.8 ↑	Serum Urine	mmol/L mmol/24 h	[210]
Randomized, double-blinded, placebo-controlled, cross-over	T2DM Hypo-Mg	n = 14	67 ± 6.0	0.75 ↑ 0.23 ↑	Serum Urine	mmol/L Mg/Cre ratio	[211]
Prospective, randomized, controlled, open-label	Type 2 DN Hypo-Mg	n = 26 n = 14	61.4 ± 7.5	0.70 ↑	Serum	mmol/L	[212]
Prospective, randomized, controlled, open-label	Type 2 DN Normo-Mg	n = 26 n = 12	61.4 ± 7.5	0.82 ↑	Serum	mmol/L	[212]
Randomized, double-blinded, placebo-controlled	IR Hypo-Mg	n = 32	43 ± 7.9	0.81 ↑	Serum	mmol/L	[214]
Randomized, double-blinded, placebo-controlled	Pre-D Hypo-Mg	n = 59	42.5 ± 9.5	0.81 ↑	Serum	mmol/L	[215]
Randomized, double-blinded, placebo-controlled	T2DM Hypo-Mg	n = 32	59.7 ± 8.3	0.74 ↑	Serum	mmol/L	[216]
Randomized, double-blinded, placebo-controlled	Grade 3 DFU Hypo-Mg	n = 29	57.2 ± 11.0	0.75 ↑	Serum	mmol/L	[217]
Observational	T1DM Hypo-Mg	n = 20	11.2 ± 3.41	0.80 ↑	Serum	mmol/L	[218]
Controlled	T2DM Hypo-Mg	n = 60	N/A	0.71 ↑	Serum	mmol/L	[219]
Randomized, double-blinded, placebo-controlled	T2DM	n = 25	63 ± 8.2	0.82 ↑ 2.47 → 5.5 ↑	Plasma RBC Urine	mmol/L mmol/L mmol/24 h	[220]
Randomized, double-blinded, cross-over	T1DM	n = 29	43 ± 2.0	0.80 ↗ 3.69 ↑	Plasma Urine	mmol/L mmol/24 h	[221]
Randomized, double-blinded, cross-over	T2DM	n = 27	61 ± 2.0	0.79 ↑ 3.35 ↑	Plasma Urine	mmol/L mmol/24 h	[221]
Randomized, placebo-controlled	T2DM	n = 26	63 ± 5.0	0.82 → 1.85 →	Serum RBC	mmol/L mmol/L	[222]
Randomized, double-blinded, placebo-controlled	T2DM	n = 25	64 ± 8.0	N/A →	Serum	mmol/L	[223]
Randomized, double-blinded, placebo-controlled, cross-over	T2DM	n = 56	52.84 ± 8.42	0.95 →	Serum	mmol/L	[224]
Randomized, double-blinded, cross-over	T2DM	n = 4	73 ± 2.5	0.89 ↑ 2.27 ↑	Plasma RBC	mmol/L mmol/L	[225]
Controlled	T2DM	n = 30	71.1 ± 6.1	0.49 ↑ * 0.93 → **	Serum Serum	mmol/L mmol/L	[226]
Randomized, double-blinded, placebo-controlled, cross-over	T2DM	n = 28	28–84	N/A → N/A →	Serum RBC	mmol/L mmol/L	[227]
Randomized, double-blinded, placebo-controlled	T2DM	n = 25	46.76 ± 6.93	0.89 → 5.99 ↑	Serum Urine	mmol/L mg/dL	[228]
N/A	T2DM	n = 9	51.6 ± 2.6	0.95 ↑ 9.2 ↑	Serum Urine	mmol/L mg/dL	[229]
Randomized, double-blinded, placebo-controlled	Type 2 DN	n = 40	41.2 ± 8.8	0.99 →	Serum	mmol/L	[231]
Randomized controlled clinical trial	T2DM	n = 20	35–60	0.85 ↑	Serum	mmol/L	[232]
Randomized, double-blinded, placebo-controlled	T2DM with grade 3 DFU	n = 35	61.1 ± 11.1	0.95 ↑	Serum	mmol/L	[233]
Randomized, double-blinded, placebo-controlled	DHD	n = 27	58.8 ± 10.1	0.99 ↑	Serum	mmol/L	[234]
Randomized, double-blinded, placebo-controlled	GDM	n = 30	30.1 ± 5.9	0.90 ↑	Serum	mmol/L	[235]
Randomized, double-blinded, placebo-controlled	T2DM and CHD	n = 27	61.7 ± 9.4	0.86 ↑	Serum	mmol/L	[236]
Randomized, cross-over	T2DM	n = 8	72.2 ± 2.0	0.86 ↑ 2.03 ↑	Plasma RBC	mmol/L mmol/L	[237]
Randomized, double-blinded, placebo-controlled	GDM	n = 20	27.8 ± 3.4	0.78 ↑	Serum	mmol/L	[238]
Randomized, double-blinded, placebo-controlled	IR	n = 25	30–70	0.922 ↗ 1.920 → 0.608 ↑	Serum RBC WB	mmol/L mmol/L mmol/L	[240]
Randomized, double-blinded, cross-over	T2DM	n = 8	59–72	0.88 ↑ 2.08 ↑	Plasma RBC	mmol/L mmol/L	[242]

CHD: coronary heart disease; Cre: creatinine; DFU: diabetic foot ulcer; DHD: diabetic hemodialysis patients; DN: diabetic nephropathy; GDM: gestational diabetes; HBP: hypertension; Hypo-Mg: hypomagnesemia; IR: insulin resistance; MonoNC: mononuclear cells; Normo-Mg: normomagnesemia; Pre-D: prediabetes; RBC: erythrocytes; TP: total protein; T2DM: Type 2 diabetes mellitus; WB: whole blood; N/A: not available. ↑: increase; ↗: trend towards an increase; → without changes. *, ** Mg ion and total Mg, respectively.

## Data Availability

No new data were created or analyzed in this study. Data sharing is not applicable to this article.
